# Impacts of sea level rise on the world’s shorebirds

**DOI:** 10.1007/s10531-026-03431-8

**Published:** 2026-08-10

**Authors:** Jennifer M.T. Magel, Scott Wilson, Tara G. Martin

**Affiliations:** 1 Department of Forest & Conservation Sciences, University of British Columbia, Vancouver, BC V6T 1Z4 Canada; 2 Wildlife Research Division, Environment and Climate Change Canada, University of Alberta, Edmonton, AB T6G 2E9 Canada

**Keywords:** Climate change, Coastal squeeze, Coastal wetlands, Habitat loss, Migratory birds, Waders

## Abstract

Sea level rise (SLR) poses a significant threat to coastal ecosystems worldwide. Migratory shorebirds rely heavily on nearshore and intertidal ecosystems throughout the annual cycle and are currently undergoing global declines due to the loss of coastal habitat, suggesting that these species may be highly susceptible to future SLR. However, the effects of SLR on shorebird habitat—and, by extension, populations—remain poorly understood. Herein, we conduct the first systematic review of SLR impacts on the world’s shorebirds. The effects of SLR across all stages of the annual cycle are expected to be predominantly negative, with 78% of habitat-level studies and 89% of population- and individual-level studies documenting overall negative impacts. Declines in suitable habitat with future SLR have been projected for several sites of international and hemispheric importance to shorebirds, and impacts on shorebird populations are generally more severe than would be projected through habitat loss alone. However, maintenance or expansion of nesting and foraging habitat appears possible in certain areas where inland movement of coastal wetlands is unimpeded by anthropogenic development. Despite an increased focus on the impacts of SLR on shorebirds over the past two decades, the number of studies remains relatively small (*n* = 37), and considerable knowledge gaps remain during the migratory period and throughout the Global South. Our findings highlight the threat of SLR to the world’s shorebirds and underscore the urgency of research on feasible adaptation and mitigation options to avoid the loss of coastal wetlands and their associated biodiversity.

**Supplementary Information:**

The online version contains supplementary material available at 10.1007/s10531-026-03431-8.

## Introduction

Climate change is increasingly considered to be one of the most pervasive threats to the persistence of coastal marine biodiversity (Hoegh-Guldberg and Bruno 2010), and climate change-associated sea level rise (SLR) poses serious risks to coastal ecosystems worldwide (Blankespoor et al. 2014; Schuerch et al. 2018; Oppenheimer et al. 2019). The Intergovernmental Panel on Climate Change projects an average rise in global sea level of 0.38–0.77 m by 2100 (Fox-Kemper et al. 2021); however, the severity of local- and regional-scale impacts may vary widely depending on factors such as coastal topography, sediment supply, vertical land movement, levels of shoreline development, and local management (Cazenave and Le Cozannet 2013; Oppenheimer et al. 2019). Feedbacks between inundation and accretion—where increased sea level and longer submersion times lead to greater sediment deposition, thereby facilitating accretion of the intertidal surface—have allowed coastal ecosystems such as salt marshes and tidal flats to keep pace with historic rates of SLR in many areas through the process of vertical accretion (Morris et al. 2002; Kirwan and Megonigal 2013). As saltwater influence extends further inland and upstream, coastal wetlands can also retreat landward into adjacent upland areas through a process known as lateral migration. However, acceleration of global SLR may overwhelm the ability of these habitats to keep pace naturally with rising sea levels into the future (Crosby et al. 2016; Saintilan et al. 2023), threatening the persistence of these highly biodiverse systems.

Impacts of SLR on coastal species and ecosystems may manifest in several ways (FitzGerald et al. 2008; Oppenheimer et al. 2019). Coastal squeeze—where intertidal habitat is squeezed between the rising sea and an inland barrier—is a serious concern along many developed coastlines, where anthropogenic barriers constrain the ability of ecosystems to shift with SLR (Pontee 2013). As human coastal populations continue to expand—with over 1 billion people living within 10 km of the coast as of 2018 (Cosby et al. 2024)—barriers such as shoreline armouring, residential and industrial infrastructure, and coastal roads have become more prevalent, exacerbating the issue of coastal squeeze (Lansu et al. 2024). Loss of coastal habitat may also occur in regions absent of such barriers if ecosystems are unable to keep pace with SLR due to factors such as local subsidence or insufficient sediment supply (Kirwan et al. 2010). For species that forage along coastal margins, the quality and availability of food resources such as benthic invertebrates may be impacted by SLR-driven changes in salinity, wave energy, and sediment distribution (Fujii 2012). Further degradation of coastal systems may also be driven by increases in the frequency of extreme sea level events (extreme tides, storm surges, and waves) that are expected with future SLR (Vitousek et al. 2017; Fox-Kemper et al. 2021). As coastal ecosystems provide vital habitat for a variety of taxa, including commercially and traditionally important fish species and millions of migratory waterbirds (Perillo et al. 2019), the potential degradation of these systems by future SLR poses a significant threat to a multitude of species.

Shorebirds (also known as ‘waders’) are one such species group threatened by SLR. Shorebirds comprise a diverse and highly threatened group of ~ 215 species within the order Charadriiformes, the majority of which rely on a mosaic of coastal habitats during one or more stages of the annual cycle (Burger 1984; Colwell 2010). Most shorebirds overwinter on the coast, and approximately 39% breed in coastal habitats (primarily on sandy beaches and salt marsh; Burger 1984). Shorebirds also rely heavily on networks of high-quality coastal staging/stopover sites during migration. These sites—which are often characterized by large expanses of intertidal mudflats—provide shorebirds with energy-rich food resources and serve as places for individuals to rest and refuel before undertaking long-distance flights (Myers et al. 1987; Warnock et al. 2001). Shorebirds undertake some of the longest migratory journeys in the world, and the loss of intertidal ecosystems and coastal food resources due to human activity has been associated with declines in shorebird populations along multiple flyways, emphasizing the importance of intact coastal networks for these species (Baker et al. 2004; Studds et al. 2017; Murray et al. 2018). Although the threat of SLR to shorebird habitat and populations has been recognized (e.g., Galbraith et al. 2002; Durell et al. 2006; Iwamura et al. 2013), the impacts of SLR on shorebirds are still not well understood and are rarely considered in species conservation planning.

While an increasing number of studies are investigating the impacts of SLR on shorebird habitat and populations, no comprehensive review of SLR impacts on shorebirds has been conducted to date. Although responses of shorebirds to SLR may be highly context-dependent, gaining a general understanding of the possible magnitude and nature of past and future impacts may help to inform decision-making regarding both species-specific management and coastal adaptation more broadly. For example, recognizing the main pathways through which SLR may influence shorebirds can help to identify possible intervention points and inform effective conservation strategies, while understanding the likelihood of habitat expansion vs. contraction with future SLR can help determine the importance of accommodation space (i.e., the area available for wetland expansion) within coastal planning. This knowledge is particularly important given the wide-ranging implications of SLR: unlike threats such as coastal development—where impacts are typically confined to a particular site—SLR is a global threat that will impact all coastal sites throughout a species’ range, potentially leading to compounding effects throughout the annual cycle. Thus, this paper represents a first attempt to summarize our current understanding of the implications of long-term SLR for the world’s shorebirds. More specifically, we aim to (1) quantify general trends in the existing body of literature on SLR impacts to shorebirds through a systematic literature review; (2) summarize the available information regarding the direction and magnitude of SLR impacts to shorebirds; (3) discuss possible pathways through which SLR may impact shorebirds during each stage of the annual cycle; and (4) identify prominent knowledge gaps and areas for future research within this field.

## Systematic literature review

### Methods

**Fig. 1:**
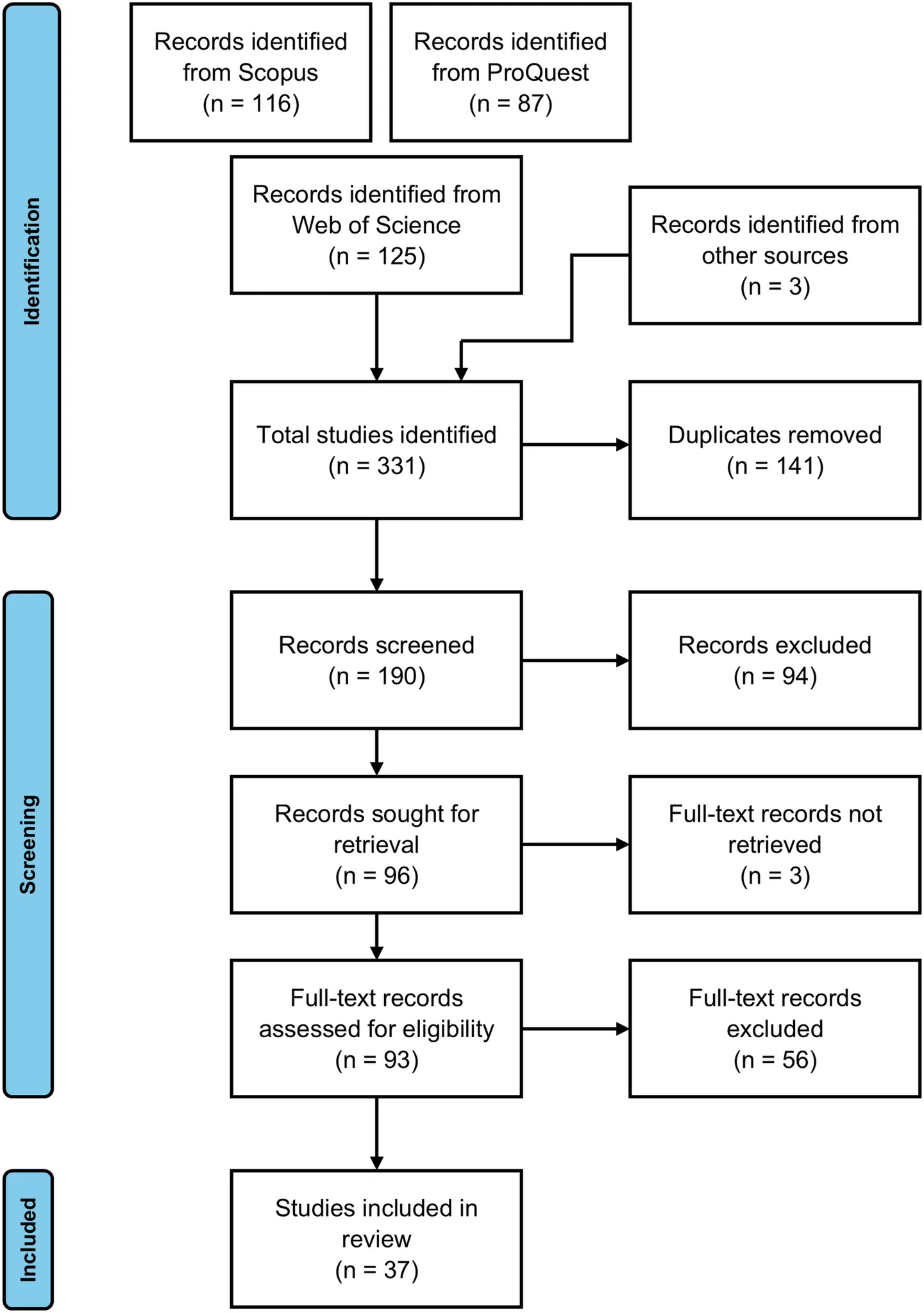
PRISMA (Preferred Reporting Items for Systematic Reviews and Meta-Analyses) diagram depicting steps taken during the systematic literature review to identify papers quantifying impacts of sea level rise (SLR) on shorebirds. Studies were excluded if they did not examine impacts of SLR (e.g., papers that focused on the response of shorebirds to storms or other extreme sea level events), did not focus on shorebirds (e.g., papers that reported impacts solely on non-shorebird species such as terns or rails), or did not provide original quantitative data to quantify SLR impacts

To gather information on the past and future impacts of SLR on shorebirds, we conducted a systematic literature search (Foo et al. 2021) using three database search engines: Web of Science, Scopus, and ProQuest. To gain a comprehensive overview of all possible SLR impacts, we limited search terms to the following: (shorebird* OR wader*) AND (“sea level rise” OR “sea-level rise”). Search settings varied slightly according to the conventions of each search engine; please see the Supplementary Methods (Supplementary Material 1) for full details on individual searches. We conducted the initial searches between 04 September 2024 and 07 October 2024 and updated each search on 09 July 2026; these searches resulted in 187 studies (following removal of duplicates) that were systematically evaluated for inclusion in the review (Fig. 1, Supplementary Material 2). In addition to those sources located through the systematic literature search, we included a further three studies previously located through ad hoc searches that were known to include data on SLR impacts to shorebirds (Durell et al. 2007; Gieder 2015; Klingbeil et al. 2021).

After identifying studies through our search criteria, we screened the title and abstract of each paper (*n* = 190) to determine whether the study documented impacts of SLR on shorebirds. For studies that were deemed relevant, we then examined the full text where available (*n* = 93) to determine whether the study provided original quantitative data examining the impact of SLR on shorebird habitat or populations/individuals. No prior reviews of SLR impacts on shorebirds were identified during this process. For the 37 studies that met our inclusion criteria, we extracted the following data: year of publication, start and end dates of the study, study length, country, region/site, flyway, species, stage of the annual cycle (breeding, migration, non-breeding, or a combination thereof; alternatively, ‘resident’ for species that were non-migratory or ‘full annual cycle’ for studies that examined impacts at the flyway scale), type of impact examined (e.g., impacts on habitat extent, habitat quality, abundance, survival, etc.), habitat response (i.e., dynamic or static depending on whether or not habitat was allowed to shift with future SLR), and overall direction of impact (positive, negative, mixed). Please see the Supplementary Methods (Supplementary Material 1) for further details regarding the screening and classification process. All publications included in the systematic review can be found in Table S1 (Supplementary Material 1), and a complete list of evaluated studies is provided in Supplementary Material 2. Extracted data and code are available at 10.5281/zenodo.21462183.

### Results

**Fig. 2:**
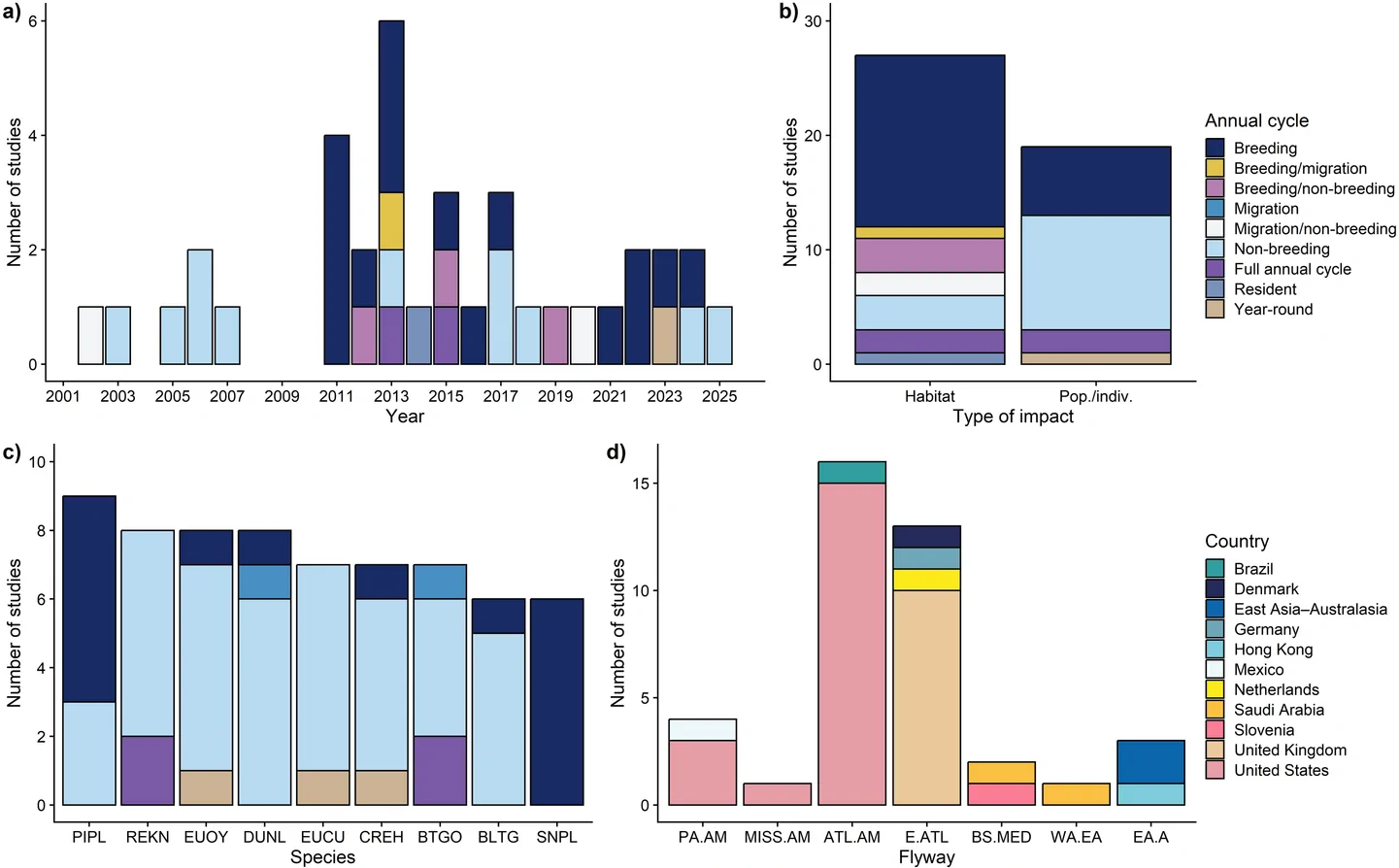
Number of studies published to date examining impacts of sea level rise on the world’s shorebirds, divided by (**a**) year, (**b**) type of impact examined, (**c**) species (only the nine most studied species are shown), and (**d**) flyway. For (b), Pop./Indiv. = populations/individuals. For (c), PIPL = Piping plover, REKN = Red knot, EUOY = Eurasian oystercatcher, DUNL = Dunlin, EUCU = Eurasian curlew, CREH = Common redshank, BTGO = Bar-tailed godwit, BLTG = Black-tailed godwit, and SNPL = Snowy plover. Species are arranged by total number of studies followed by number of studies conducted during the non-breeding period. For (d), PA.AM = Pacific Americas, MISS.AM = Mississippi Americas, ATL.AM = Atlantic Americas, E.ATL = East Atlantic, BS.MED = Black Sea–Mediterranean, WA.EA = West Asian–East African, and EA.A = East Asian–Australasian. For countries, ‘East Asia–Australasia’ refers to Iwamura et al. (2013) and Nicol et al. (2015), two studies that quantified impacts of sea level rise on stopover habitat across eight countries (USA [Alaska], Russia, Japan, Malaysia, North Korea, South Korea, Australia, New Zealand) and four regions (Yellow Sea, East China Sea, Southeast Asia, Malay Archipelago) along the East Asian–Australasian Flyway

Our search yielded 37 studies explicitly quantifying SLR impacts on shorebirds, all of which were published between 2002 and 2025 (Fig. 2a). All studies except for two (Laursen et al. 2023; Santos et al. 2025) predicted impacts of future SLR, as opposed to examining past changes. Approximately half of future-focused studies (17/35, 48.6%) examined SLR impacts up to the year 2100, simulating changes in shorebird habitat and/or populations over an average period of 90.9 ± 7.0 (mean ± SD) years. However, endpoints for all included future-focused studies ranged from 2030 to 2125, with study length ranging from 30 to 139 years (mean = 76.7 ± 27.8 years). Seven studies did not examine SLR impacts over a defined time period, instead predicting the impacts of particular levels of SLR (e.g., every 0.5 m up to 3 m) without establishing a specific endpoint for the study (Table S1).

The majority of published studies have examined impacts during the breeding (23/37, 62.2%) or non-breeding (19/37, 51.4%) periods, with relatively few (6/37, 16.2%) having quantified impacts at staging and stopover sites used during migration (note that some studies examined impacts during multiple stages of the annual cycle, so totals exceed 100%) (Fig. 2a). Only one study focused on a non-migratory species (Black oystercatcher (*Haematopus bachmani*) in Oregon, USA; Hollenbeck et al. 2014), and only two studies examined impacts of SLR across the entire annual cycle, both of which were conducted across the East Asian–Australasian Flyway (Iwamura et al. 2013; Nicol et al. 2015).

Approximately three-quarters of studies (27/37, 73.0%) quantified indirect impacts of SLR on shorebirds via changes in their habitat, while just over half (19/37, 51.4%) examined impacts to shorebird populations or individuals (including nine studies that quantified impacts to both habitat and populations/individuals) (Fig. 2b). Studies examining impacts to habitat primarily quantified changes in habitat extent (26/27, 96.3%). Studies quantifying impacts to populations/individuals examined a more diverse range of metrics, the most common being overwinter survival (6/19, 31.6%) and abundance (4/19, 21.1%) (Table S1). Please see the Supplementary Methods (Supplementary Material 1) for a complete list of habitat- (*n* = 13) and population/individual-level metrics (*n* = 17) examined in the studies. Impacts of SLR have been examined for 29 species of shorebirds from four families: Scolopacidae (i.e., sandpipers and allies; 18 spp.), Charadriidae (i.e., plovers and allies; 7 spp.), Haematopodidae (i.e., oystercatchers; 3 spp.), and Recurvirostridae (i.e., stilts and avocets; 1 sp.) (Table S1). The seven most studied species (appearing in 7–9 studies each) were the Piping plover (*Charadrius melodus*), Red knot (*Calidris canutus*), Eurasian oystercatcher (*Haematopus ostralegus*), Dunlin (*Calidris alpina*), Eurasian curlew (*Numenius arquata*), Common redshank (*Tringa totanus*), and Bar-tailed godwit (*Limosa lapponica*) (Fig. 2c, Table S1).

**Fig. 3:**
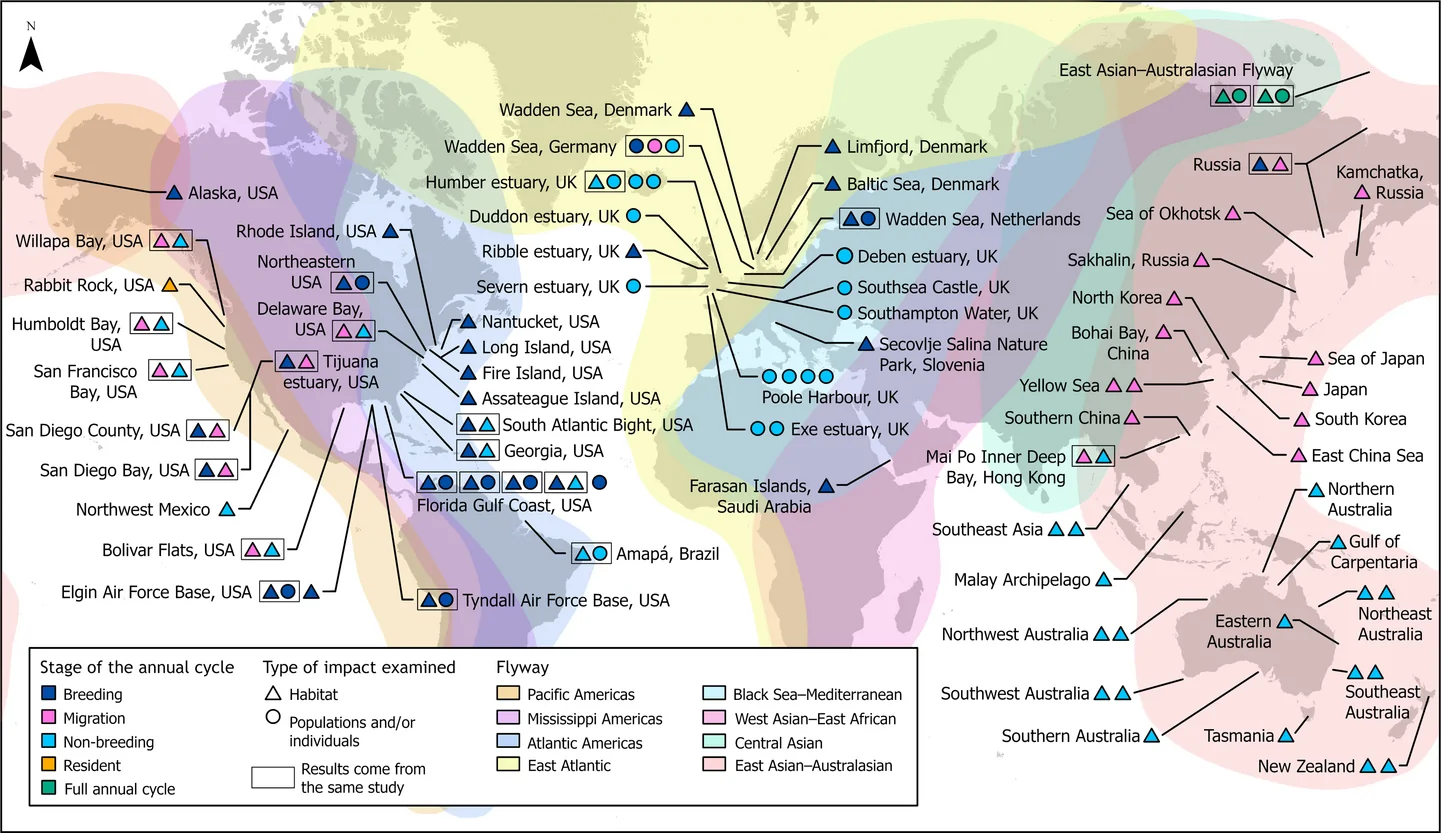
Location of all sites for which the impacts of sea level rise on shorebirds have been quantified. Sites are categorized according to the stage of the annual cycle and the type of impact examined. For individual sites, results that come from the same study are indicated through the use of rectangular boxes (e.g., a single study was conducted at Bolivar Flats, USA, which examined impacts on habitat used by both migrating and non-breeding shorebirds). Note that the number of sites examined within a particular country or region is not necessarily representative of the number of individual studies conducted in that region (e.g., results for sites within the East Asian–Australasian Flyway region come from only three studies). For the number of published studies conducted in each country, see Fig. 2d. Flyway boundaries from Boere and Stroud (2006)

Finally, seven of the eight shorebird flyways were represented in our systematic review, with over three-quarters of studies focusing on either the Atlantic Americas Flyway (16/37, 43.2%) or the East Atlantic Flyway (13/37, 35.1%), followed by the Pacific Americas Flyway (4/37, 10.8%) (Fig. 2d). None of the identified studies examined SLR impacts along the Central Asian Flyway. Sea level rise impacts have been quantified in 17 countries and four wider regions (Yellow Sea, East China Sea, Southeast Asia, Malay Archipelago) across these seven flyways (Fig. 3), with nearly three-quarters of studies conducted in either the United States (17/37, 45.9%) or United Kingdom (10/37, 27.0%) (Fig. 2d). All other countries/regions were the focus of only one to two studies, and we did not locate any studies conducted within Africa.

## Summary of the impacts of sea level rise on shorebirds

**Fig. 4:**
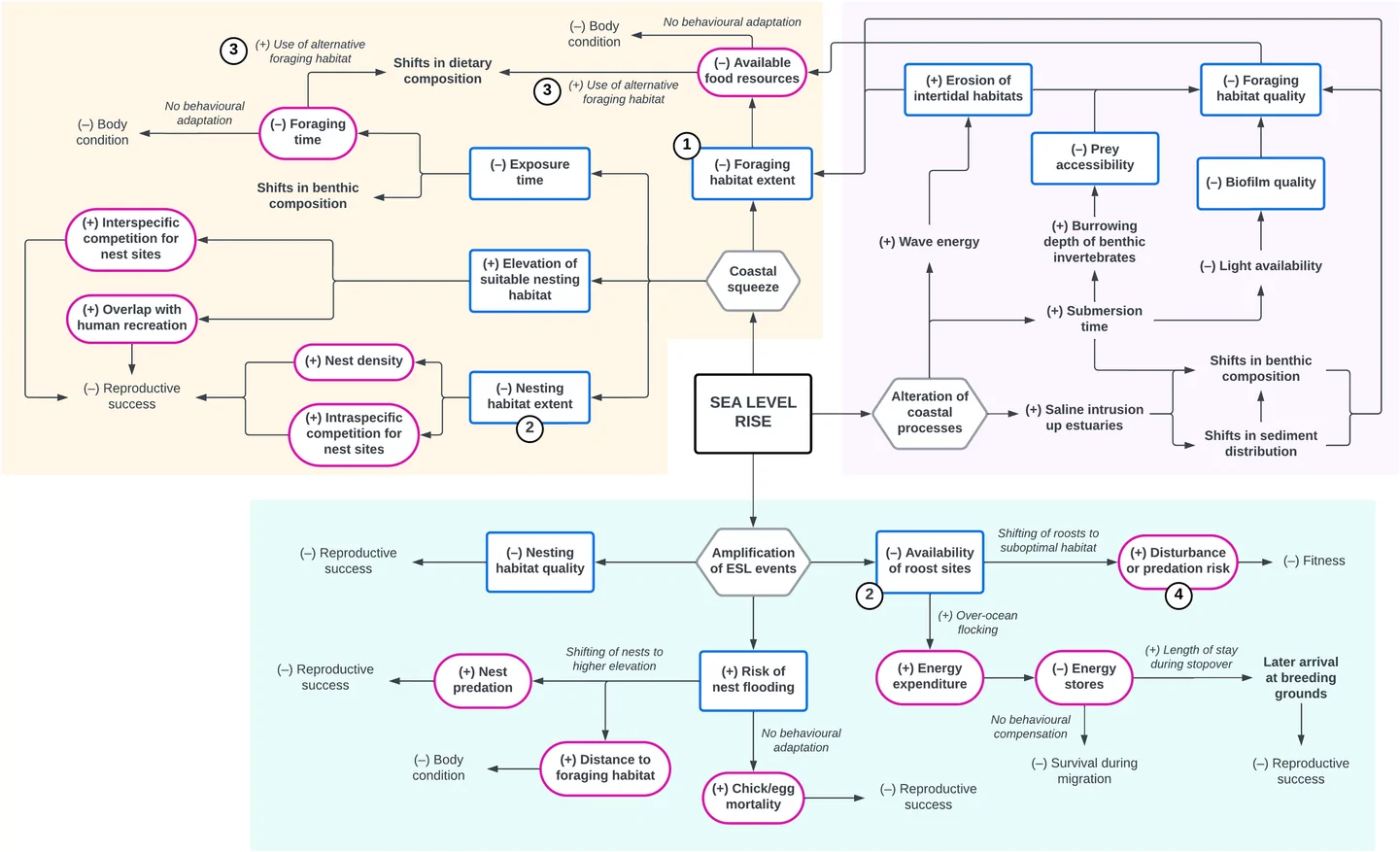
Summary of the possible impacts of future sea level rise (SLR) on the world’s shorebirds across all stages of the annual cycle. While not a direct result of SLR, the impacts of extreme sea level (ESL) events on shorebirds are also depicted here, as SLR is expected to increase the frequency of such events. Impacts have been grouped into three broad categories, depicted by the shaded boxes, according to the primary mechanism driving each pathway (i.e., coastal squeeze, alteration of coastal processes, amplification of ESL events). Impacts to shorebird habitat are depicted using the blue rectangles, impacts to individuals/populations are depicted using the pink ovals, and possible behavioural adaptations are included in italics. (+) refers to increases in the associated behaviour or phenomenon, while (–) refers to decreases. Numbers correspond to specific impacts illustrated in Fig. 5

**Fig. 5:**
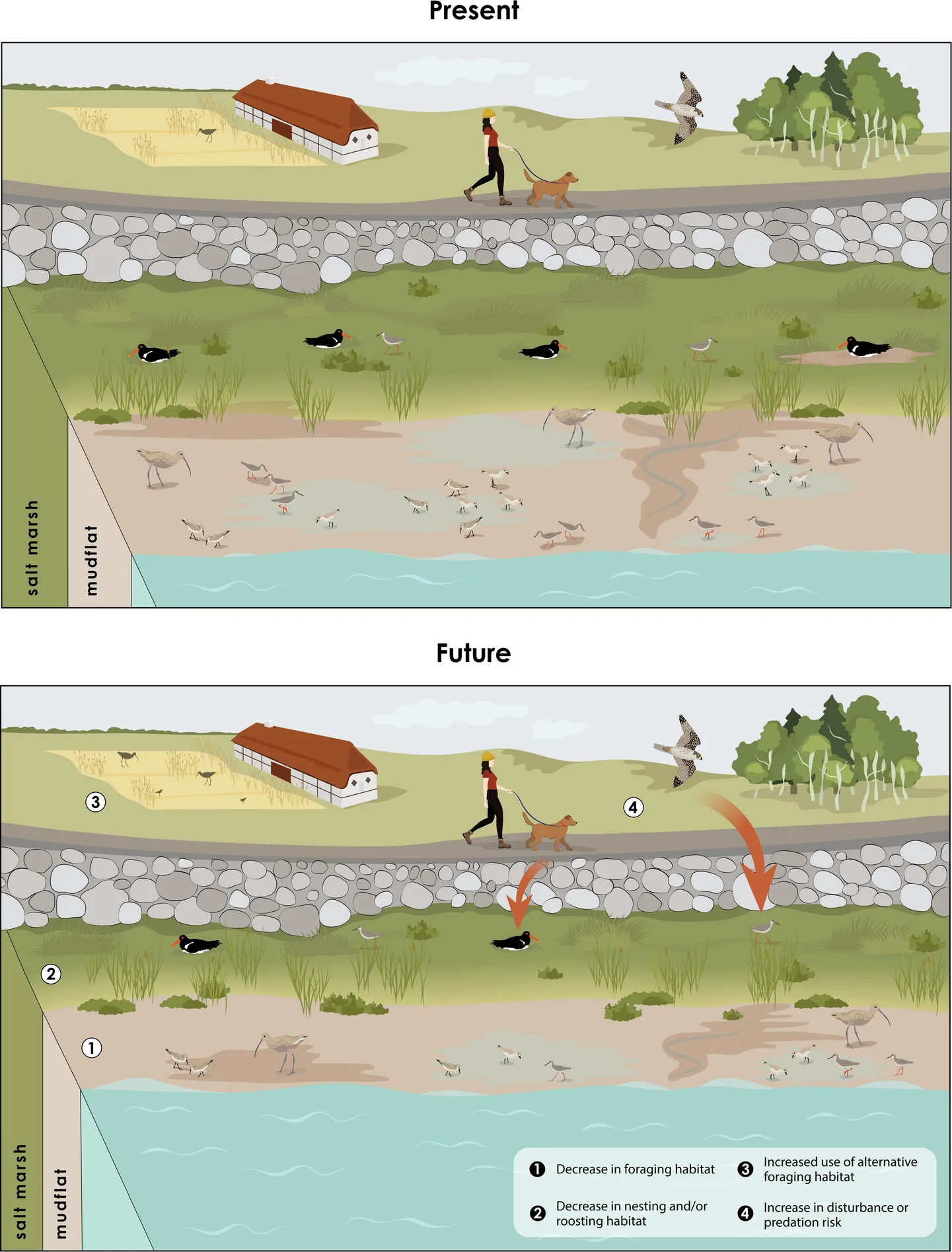
Possible impacts of future sea level rise (SLR) and resulting coastal squeeze on tidal flat and salt marsh habitats used by shorebirds. The top panel depicts present-day conditions prior to SLR, while the bottom panel illustrates possible impacts in areas where inland migration of coastal wetlands is prevented by artificial barriers. Four potential impacts are depicted: (1) declines in the extent of tidal flat foraging habitat (and associated declines in numbers of foraging shorebirds), (2) declines in the extent of salt marsh nesting and roosting habitat (and associated declines in numbers of nesting/roosting shorebirds), (3) increased use of alternative foraging habitats, and (4) increased exposure to predation or human disturbance as shorebirds are pushed further inland. Please see Fig. 4 for a more detailed depiction of the possible mechanisms and pathways leading to each of the above impacts, their potential population- or individual-level effects, and additional pathways through which SLR may impact shorebirds

**Fig. 6:**
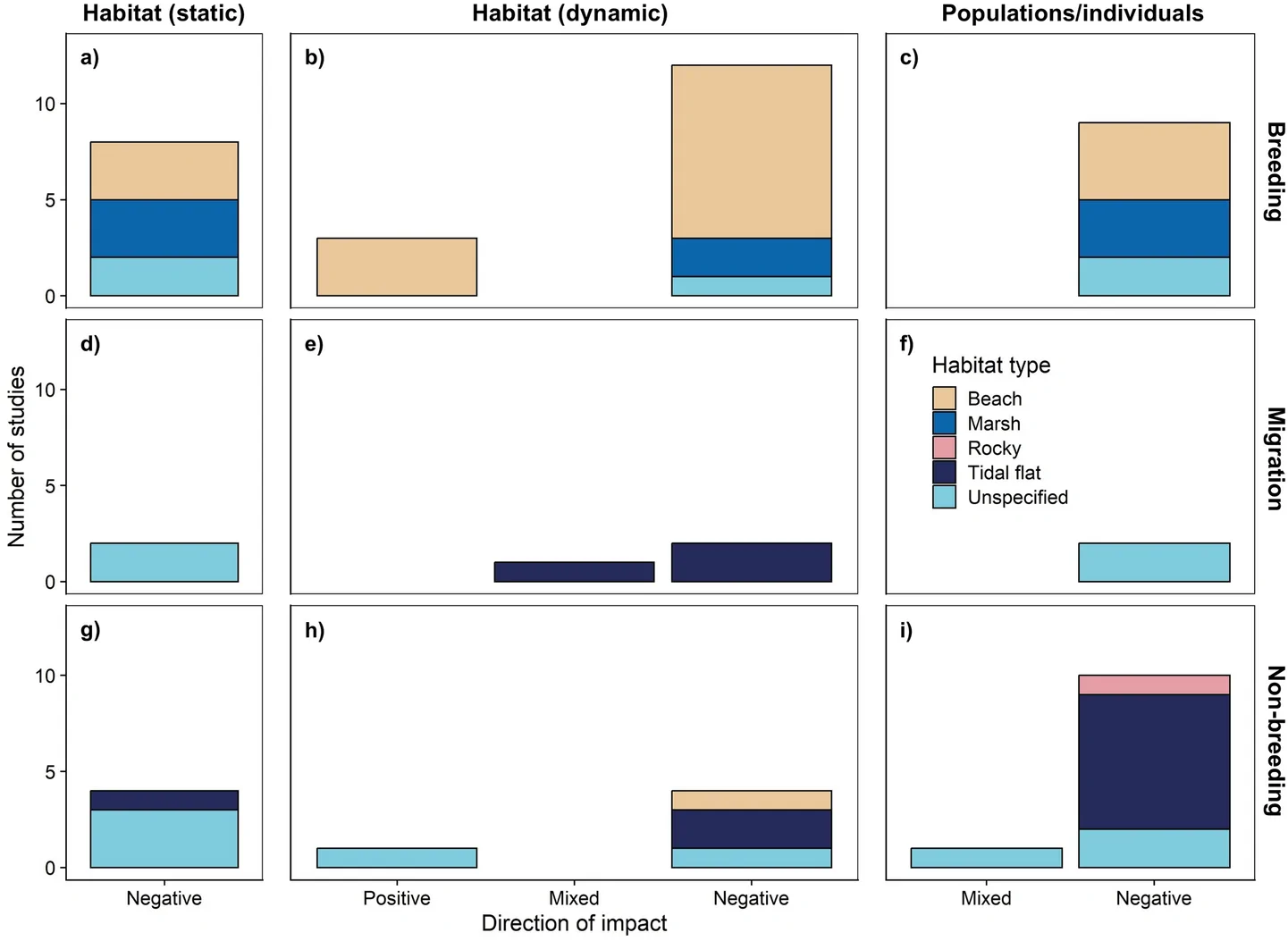
Number of studies reporting positive, negative, and mixed impacts of future sea level rise (SLR) on the world’s shorebirds, divided by stage of the annual cycle (top = breeding; middle = migration; bottom = non-breeding) and type of impact (left = habitat (static); center = habitat (dynamic); right = shorebird populations/individuals). ‘Static’ refers to studies that did not account for the ability of habitat to shift with future SLR, while ‘dynamic’ refers to studies that allowed for the possibility of future habitat expansion. Two studies examining past impacts of SLR (Laursen et al. 2023; Santos et al. 2025) were excluded from this figure

Past and future impacts of SLR on the world’s shorebirds are highly diverse (Figs. 4 and 5) and include impacts on the breeding, migratory, and non-breeding grounds. The effects of SLR across all stages of the annual cycle are predominantly negative (Fig. 6, Table S3), with 77.8% (21/27) of habitat-level studies and 89.5% (17/19) of population- or individual-level studies documenting overall negative impacts. Future declines in suitable habitat were projected across several habitat types (including beach, rocky intertidal, salt marsh, and tidal flat) and for many sites of international or hemispheric importance to shorebirds, including San Francisco and Humboldt Bays (United States), the Colorado River Delta and Marismas Nacionales (Mexico), Mai Po Inner Deep Bay (Hong Kong), and the Yellow Sea region in East Asia. Expansion of shorebird nesting or foraging habitat with SLR was reported for one or more sites in approximately one-third of studies (10/27; 37.0%); however, this was often only for particular SLR scenarios (usually high SLR of 1–2 m by 2100). Although we did not extract data on the magnitude of SLR impacts for each study, studies that examined impacts of SLR on both shorebird habitat and populations documented disproportionately severe population-level impacts compared to habitat impacts in almost all (8/9) cases (i.e., the expected % change in shorebird populations was larger than the % change in habitat extent). Impacts of SLR on shorebird habitat and populations/individuals during each stage of the annual cycle are discussed in more detail below, along with potential pathways through which SLR may impact shorebirds during each stage.

### Changes in the extent of suitable habitat

#### Breeding

Studies examining SLR impacts to shorebird breeding habitat (*n* = 20) focused primarily on sandy beach and salt marsh ecosystems. Most studies examined impacts on beach-nesting species (13/20, 65.0%), with all but two of these studies taking place along the Atlantic and Gulf coasts of the United States (11/13, 84.6%). Despite this limited geographical scope, predicted changes in beach extent resulting from future SLR were highly variable. Most studies predicted declines in beach nesting habitat (Fig. 6a, b), with projected losses ranging from a low of 13% for Snowy plover (*Anarhynchus nivosus*) along the Florida Gulf Coast (Linhoss et al. 2013) to a high of 45.1% for Piping plover on Fire Island, USA (Zeigler et al. 2022). Declines in habitat were also predicted for species breeding along the coasts of Nantucket Island (McCarthy 2022) and Georgia (Hunter et al. 2015) in the United States, as well as Saudi Arabia’s Farasan Islands (AlRashidi et al. 2012). However, several studies produced mixed results, with the direction of habitat change varying either across sites (Naughton 2013) or depending on whether simulations accounted for habitat migration with SLR (Seavey et al. 2011; Sims et al. 2013) (Table S3). Only one study—which examined changes in Piping plover habitat on Assateague Island, USA—projected increases in suitable nesting habitat for all SLR scenarios examined (Gieder 2015). These changes primarily resulted from a SLR-driven decrease in herbaceous cover along focal beaches, rather than an expansion in beach area, emphasizing the importance of assessing changes in fine-scale habitat suitability alongside shifts in the extent of broader land-cover types.

As changes in beach habitat will likely be highly variable with future SLR, we expect that the direction and magnitude of impacts to shorebird populations will vary accordingly. To date, changes in populations of breeding/nesting shorebirds as a result of SLR have been assessed only for Snowy plovers nesting along the Gulf Coast of Florida (Aiello-Lammens et al. 2011; Convertino et al. 2011; Linhoss et al. 2013; Aiello-Lammens and Akçakaya 2017), making it difficult to draw conclusions about the potential impact of SLR on population-level parameters for beach-nesting shorebirds in general. All four studies predicted negative impacts of future SLR on Snowy plover populations (Fig. 6c). However, in the three studies that quantified changes in both plover populations and nesting habitat, projected changes in population metrics were disproportionately greater than projected changes in habitat extent. This suggests that the effects of habitat loss do not translate linearly into population-level impacts and emphasizes the importance of moving beyond simple studies of habitat change (discussed further below).

Studies on marsh-nesting species comprised one fifth (4/20, 20.0%) of all sources examining SLR impacts to shorebird breeding habitat. Although these studies were geographically diverse, taking place across four different countries, they all predicted declines in salt marsh nesting habitat with future SLR (Fig. 6a, b). Projected declines ranged widely in severity and included the potential loss of breeding habitat for Black-tailed godwit (*Limosa limosa*), Dunlin, and Ruff (*Calidris pugnax*) along the Wadden and Baltic Sea coasts of Denmark (Clausen et al. 2013); Willet along the U.S. Atlantic coast (Klingbeil et al. 2021); Common redshank in the Ribble Estuary, UK (Harmer 2023); and Eurasian oystercatcher in the Netherlands sector of the Wadden Sea (van de Pol et al. 2024). Across all sites and SLR scenarios examined, projections of future habitat loss ranged from a low of 6% (RCP 2.6, 1986–2125; van de Pol et al. 2024) to a high of 97.3% (RCP 8.5, 2017–2100; Harmer 2023); however, all studies projected declines in habitat area of over 40% for at least one of the SLR scenarios modelled. This is alarming, considering that even minor increases in sea level can substantially impact coastal breeding shorebirds. The low-elevation nest sites preferred by many species experience the greatest risk of nest flooding, the rate of which may increase significantly with only a few cm of SLR (van de Pol et al. 2024). As such, impacts to marsh-nesting shorebirds may begin to manifest well before marsh habitat is inundated or physically transitions to tidal flat.

Changes in the extent of suitable nesting habitat with SLR have additional implications for coastal breeding shorebirds (Fig. 4). Loss of suitable habitat may lead to increased nest density or greater intraspecific competition for nest sites, with potential negative consequences for shorebird reproductive success (Seavey 2009; Catlin et al. 2019). These impacts will likely be most salient for species or individuals with high nest site fidelity that are unable to shift their nest locations to match changes in habitat availability. Alternatively, shifts in the distribution of remaining habitat towards higher elevations may increase interspecific competition with other coastal nesting species such as terns and gulls (Seavey et al. 2011). For beach-nesting shorebirds in particular, inland migration of sandy beach habitat with future SLR may lead to an increased overlap of suitable nest sites with upland areas used by humans for recreation (Seavey et al. 2011). This overlap may disrupt shorebird nesting behaviour and lead to increased egg and chick mortality (Ruhlen et al. 2003; Weston et al. 2012), thus exacerbating the issue of human–bird conflict that is already present on many nesting beaches. Consequently, coastal-breeding shorebirds may still face multiple challenges even in areas where nesting habitat is able to expand inland with SLR.

#### Migration

Despite the importance of coastal ecosystems and their associated food resources during the migratory period, relatively few studies to date (5/37, 13.5%) have quantified the impact of SLR on shorebird staging and stopover habitat. However, unlike the available literature on breeding habitat, which appears to be concentrated overwhelmingly along the two Atlantic flyways, the impacts of SLR on stopover habitat have been investigated primarily along the Pacific Americas Flyway (Galbraith et al. 2002; Naughton 2013) and the East Asian–Australasian Flyway (Iwamura et al. 2013; Nicol et al. 2015; Wikramanayake et al. 2020) (Figs. 3, S1c).

In general, SLR appears to pose a significant threat to migratory stopover habitat. Large declines in tidal flat area are projected for several stopover sites of international and hemispheric importance along both Pacific Ocean flyways, including San Francisco Bay (39.4–83.1%), Humboldt Bay (28.6–91.3%), and Willapa Bay (18.1–61.5%) in the United States (Galbraith et al. 2002); Mai Po Inner Deep Bay in Hong Kong (76–92% at high tide; Wikramanayake et al. 2020); and the Yellow Sea region in East Asia (13.3–49.2%; Iwamura et al. 2013). Increases in stopover habitat under very high SLR (1–2 m by 2100) have been predicted for a handful of sites in the United States, including Delaware Bay, Bolivar Flats, and the Tijuana Estuary (Galbraith et al. 2002; Naughton 2013); however, we expect that tidal flat expansion at these sites is unlikely to compensate for the drastic declines predicted elsewhere along the flyway under high SLR scenarios. In addition, impacts of stopover habitat loss on shorebird populations may be disproportionately greater than predicted by habitat loss alone, particularly if migratory ‘bottleneck’ sites (i.e., sites that are used by a large percentage of the population during migration) are affected (Newton 2006; Iwamura et al. 2013; Nicol et al. 2015).

While only three studies to date have evaluated impacts of SLR on shorebird populations during the migratory period (Iwamura et al. 2013; Nicol et al. 2015; Laursen et al. 2023; Fig. S1d), the implications of future habitat loss may be partially inferred by examining shorebird responses to historic tidal flat loss resulting from human development. The Yellow Sea, which serves as the main migration bottleneck for many species along the East Asian–Australasian Flyway, has undergone substantial loss of tidal flat habitat since the 1980s due to reclamation for aquaculture and coastal development (Murray et al. 2014). Loss of habitat at this key staging site has been linked to population declines for several species of shorebirds along the flyway, including the endangered Far Eastern curlew (*Numenius madagascariensis*), Great knot (*Calidris tenuirostris*), and Spoon-billed sandpiper (*Calidris pygmaea*) (Moores et al. 2016; Studds et al. 2017). The declines in Far Eastern curlew and Great knot are suspected to result from low survival following departure from the Yellow Sea region, suggesting that the loss of stopover habitat may limit the ability of shorebirds to acquire sufficient energy to power migration (Studds et al. 2017). This likely occurs through a combination of decreases in the overall resource pool and increased competition for food on the remaining tidal flats (Wang et al. 2022). Given that shorebirds primarily rely on stopover sites as a means of replenishing their energy stores, impacts of SLR during migration will likely manifest through these reductions in food supply resulting from the loss of prey-rich intertidal flats (see also 'Changes in habitat quality or suitability' below).

#### Non-breeding

Although most shorebirds overwinter on the coast, impacts of SLR on non-breeding habitat were examined in only a quarter of identified studies (10/37, 27.0%; Fig. S1e). Similar to our findings for both breeding and stopover habitat, impacts of SLR on non-breeding habitat appear to be largely negative, with 8/10 studies reporting declines in foraging habitat at wintering sites (Fig. 6g, h). Projected habitat loss ranged from a low of 2.2–11.2% in the Humber Estuary, UK (0.1–0.5 m SLR, 2003–2050; Fujii 2006) to a high of 98.1% for key sites throughout southeast Australia (3 m SLR; Iwamura et al. 2013). Large declines in non-breeding habitat were also projected for six other sites of international or hemispheric importance for shorebirds: San Francisco Bay, Humboldt Bay, and Willapa Bay in the United States (Galbraith et al. 2002; see above); the Colorado River Delta (-59%) and Marismas Nacionales (-77%) in northwest Mexico (Garcia-Walther et al. 2024); and Mai Po Inner Deep Bay in Hong Kong (Wikramanayake et al. 2020; see above). However, increases in foraging habitat for Semipalmated sandpiper (*Calidris pusilla*) have been observed in remote coastal wetlands in northeast Brazil, where tidal flat habitat has migrated inland over the past three decades into areas formerly occupied by mangroves (Santos et al. 2025). Many smaller wetlands throughout northwest Mexico are also predicted to be unaffected by future SLR, suggesting that these sites may increase in importance into the future (Garcia-Walther et al. 2024). While expansion or maintenance of smaller coastal wetlands provides some promise, the ability of these sites to act as a buffer against the impacts of long-term SLR is difficult to predict without a more comprehensive understanding of projected SLR impacts across larger regions or flyway networks.

Of the 19 studies that examined impacts of SLR on shorebird populations/individuals, over half (13/19, 68.4%) took place during the non-breeding period. Only four of these studies directly translated projected shifts in non-breeding habitat into changes in local shorebird populations, while the remaining nine studies quantified impacts of SLR on non-breeding shorebirds without directly modelling or reporting changes in habitat extent. Over three-quarters of studies on non-breeding populations (10/13, 76.9%) took place in western Europe (Fig. S1f), and approximately half (6/13, 46.2%) examined impacts on overwinter survival. Impacts of SLR on the survival of non-breeding shorebirds varied greatly according to the species, sites, and SLR scenarios examined, with predicted changes in survival ranging from 0% (i.e., no change in survival) to 100% (i.e., complete mortality). These large disparities in survival estimates were largely due to differences in habitat quality among sites, which was influenced by factors such as prey biomass (Stillman et al. 2005; Durell et al. 2007), human disturbance (Collop 2017), and the availability of alternative foraging habitats (e.g., agricultural fields; Durell et al. 2006, 2007). For example, foraging of shorebirds wintering in Poole Harbour, UK is already limited by low prey biomass and relatively short tidal flat exposure times; as such, individuals in this area are much more susceptible to decreases in foraging habitat availability resulting from SLR than conspecifics wintering in the higher-quality Exe estuary (Durell et al. 2006, 2007). Taken together, these findings emphasize the importance of site-specific research and suggest that impacts of SLR on populations of non-breeding shorebirds will depend not only on changes in the extent of intertidal habitats but also on the quality of those habitats (discussed further below) and the prevalence of supplementary feeding areas.

Given that shorebirds rely heavily on tidal flats for foraging during both the non-breeding and migratory periods, improvement of habitat quality and preservation of alternative foraging habitats may help to mediate the impacts of tidal flat loss at both wintering and stopover sites. It is also possible that inundation of foraging habitat in some areas may be offset by the expansion of tidal flats elsewhere (e.g., in areas with favourable geomorphology, low relative SLR, and little to no coastal development). Indeed, global tidal flat area—including that within Important Bird and Biodiversity Areas used by non-breeding shorebirds (Santos et al. 2023)—appears to have remained relatively stable over the past two decades, with large losses in some areas being offset by gains in others (Murray et al. 2022). Even if SLR does lead to the loss of non-breeding habitat at key wetlands (e.g., Garcia-Walther et al. 2024), greater freedom of movement during the non-breeding season (compared to the breeding and migratory periods) may assist shorebirds in adapting to changes in habitat availability during this stage of the annual cycle (Newton 2004). Shorebirds are generally distributed most widely during the non-breeding period, and the lack of an impending energy-intensive activity (such as reproduction or migration) provides individuals with greater flexibility in terms of site selection and additional time to search for an area with sufficient food resources (Newton 2004). However, the ability of shorebirds to locate and move to alternative sites may vary widely depending on differences in exploratory behaviour and site faithfulness between species and/or individuals, with highly site-faithful species or older birds less likely to explore new areas (Lourenço et al. 2016; Chan et al. 2023; Schwemmer et al. 2026). Given the limited geographical scope of our knowledge of SLR impacts during the non-breeding period, we currently lack an understanding of which sites may be ‘winners’ and which may be ‘losers’ when it comes to SLR. This information will be vital in the future to manage and prioritize coastal wetlands for shorebird conservation.

### Changes in habitat quality or suitability

In addition to driving the expansion and contraction of coastal wetlands (see 'Changes in habitat quality or suitability' above), SLR may also impact the quality of intertidal habitats used by shorebirds. These effects may be particularly salient for soft-sediment habitats such as tidal flats, where increases in water depth due to SLR can drive changes in coastal processes that influence food availability (Fujii 2006, 2012) (Fig. 4). Within estuaries, SLR may cause an upstream shift in the turbidity maximum (the zone of freshwater and saltwater mixing), leading to a redistribution of fine sediments from the wide expanse of the outer estuary to the narrow reaches of the upper estuary (Fujii 2012). These changes in salinity and sediment distribution may then drive a similar upstream shift in the distribution of benthic invertebrates to the narrower upper estuary (Fujii 2012), leading to a decrease in high-quality foraging habitat even in areas where tidal flat extent remains stable (Fujii 2006). Movement of fine sediments off the tidal flats may also be driven by increases in wave energy resulting from increased water depths (Fujii 2012). This may further reduce the suitability of tidal flats for foraging, as the ability of shorebirds to detect and capture benthic invertebrates is generally highest on mudflats characterized by fine sediments (Quammen 1982; VanDusen et al. 2012). However, some species of shorebirds prefer coarser substrates (Hill et al. 1993; VanDusen et al. 2012) and may, therefore, benefit from shifts towards sandier tidal flats. Changes in foraging habitat quality may also occur in areas where tidal flats have shifted inland with SLR into areas formerly occupied by other habitat types. For example, although tidal flats in northeast Brazil have increased in area over the past three decades due to landward expansion, the areas of newly created tidal flat host significantly fewer benthic invertebrates, resulting in limited use of these areas by shorebirds (Santos et al. 2025). As such, SLR-driven changes in the physical characteristics of tidal flats and the composition of associated invertebrate communities may impact shorebirds independently of changes in habitat extent.

In addition to changes in the physical characteristics of tidal flats, SLR-driven declines in exposure time (i.e., the amount of time for which intertidal habitats are exposed to the open air) may impact both the composition and distribution of benthic invertebrate communities and the production of biofilm. Biofilm (i.e., microphytobenthos—a thin layer of microbes, organic detritus, and sediment suspended within a mucilaginous matrix on the surface of exposed tidal flats; Characklis and Marshall 1990) is a vital food resource for many small-bodied shorebirds (Kuwae et al. 2008; Jardine et al. 2015). Decreases in exposure time and light availability with increasing water levels may limit photosynthesis by diatoms (Mitbavkar and Anil 2004), one of the major energy-dense components of biofilm (Schnurr et al. 2019; Ollinik et al. 2021), leading to changes in biofilm quality. Increases in water depth and submersion time may also translate into less abundant and diverse invertebrate communities on remaining tidal flats (Paavo et al. 2011), although growth of larger taxa such as bivalves may benefit from decreases in exposure time (Wanink and Zwarts 1993), potentially benefitting larger shorebirds (e.g., oystercatchers) that prey on these species. Finally, increased submersion time and shifts towards sandier tidal flats may lead to increases in the burrowing depth of benthic invertebrates (Esselink and Zwarts 1989; de Goeij and Honkoop 2002), potentially decreasing prey accessibility. Although few studies identified through our review explicitly quantified changes in food availability with SLR, decreases in exposure time and the resulting impacts on intertidal habitat will occur anywhere that coastal wetlands contract due to SLR. While evidence for population limitation due to food availability is scarce for shorebirds, declines in food resources at stopover sites have had population-level consequences for some species (e.g., Red knot feeding on horseshoe crab eggs in Delaware Bay; Baker et al. 2004), suggesting that declines in habitat quality may have serious implications for shorebird populations.

### Changes in habitat availability

Aside from its impact on food availability, changes in the exposure time of intertidal habitat may also threaten shorebirds through decreases in total foraging time. Shorebird foraging in intertidal areas is heavily influenced by the tidal cycle, with the majority of foraging occurring during low tides when the greatest area of intertidal habitat is exposed (Burton et al. 2004; Fonseca et al. 2017). Decreases in exposure time with SLR may constrain the availability of these vital habitats, with foraging grounds being exposed and accessible to shorebirds for progressively shorter periods of time as sea levels rise (Nightingale et al. 2018). As foraging time is a key factor influencing the ability of shorebirds to meet their daily energy requirements and successfully refuel during migration (Evans 1976), species that are unable to adapt to decreases in available foraging time (e.g., through increased use of alternative foraging habitats; Fig. 5) may suffer declines in survival, reproduction, and migratory success. Despite this, very few studies identified through our review attempted to quantify changes in exposure time and/or foraging time with future SLR (but see Collop 2017; Nightingale et al. 2018), suggesting the need for further research on this topic. Moving beyond a focus on habitat area to consider the ways in which SLR will impact available foraging time will provide a deeper understanding of the extent to which shorebirds may be impacted by future SLR.

## Insight from research on the impacts of extreme sea level events

In addition to the slow, decadal-scale changes in habitat that are expected to occur with long-term SLR, increases in sea level may have more acute impacts on shorebirds through their interaction with extreme sea level (ESL) events—abnormally high water levels resulting from extreme tides, storm surges, and waves (Gregory et al. 2019). Sea level rise is expected to magnify the frequency of ESL events, with historically rare ESL events becoming an annual occurrence in many coastal areas by 2050 (Oppenheimer et al. 2019). Despite regional variations in the magnitude of relative SLR, even moderate increases in sea level may disproportionately increase the occurrence of ESL events and resulting coastal flooding (Vitousek et al. 2017; Taherkhani et al. 2020), with the potential for particularly severe impacts in cases where storm surge occurs in conjunction with spring or king tides (McLaughlin et al. 2022). Thus, although ESL events are not a direct product of SLR—and studies focusing on such events were, therefore, largely excluded from our systematic review—SLR may exacerbate the impact of these events on shorebirds across all stages of the annual cycle. While this paper does not aim to explore all these impacts in detail, we note some of these findings below.

### Impacts of ESL events on nesting shorebirds

For coastal-breeding shorebirds, increases in ESL events may heighten the risk of nest flooding and alter the quality and availability of suitable nesting habitat, with potential consequences for shorebird reproductive success (Fig. 4). Inundation of nest sites may decrease reproductive output by washing away eggs and chicks (van de Pol et al. 2010) or by damaging eggs and preventing them from hatching (Que et al. 2015). Flood risks have increased for many species over the past several decades (van de Pol et al. 2010; Koivula et al. 2025), and flooding-induced declines in reproductive success have already led some populations to become unstable (e.g., Eurasian oystercatcher breeding in the Wadden Sea; van de Pol et al. 2010). The ability of shorebirds to adapt their nest site selection in response to increased flood risk also appears limited due to the unpredictable nature of flood events and the trade-offs (in food availability, predation risk, etc.) inherent in nesting at higher elevations (Bailey et al. 2017; Plaschke et al. 2019), although some conservation projects are attempting to decrease nest loss due to storm surges by gradually moving nests up the shoreline (e.g., Moore and Williams 2005). Projections of nest flooding under future SLR appear dire for areas already subject to hurricanes and severe storms (Seavey et al. 2011), suggesting that ESL-related flooding events may pose a substantial threat to coastal-breeding shorebirds and potentially signalling the need for increased conservation intervention.

Extreme sea level events may also lead to changes in the suitability of coastal areas for nesting. For beach-nesting species specifically, the quality of nesting habitat in sandy environments is strongly linked to beach morphology (de Alegría-Arzaburu et al. 2025), which may change dramatically following re-shaping by storm events and other coastal processes. Thus, increases in the frequency of ESL events with future SLR may cause the availability of once-reliable nesting habitats to become unpredictable, likely compromising the reproductive success of site-faithful species. Conversely, human attempts to mitigate the impacts of increased coastal flooding (e.g., through construction of artificial barriers) may also negatively impact coastal-breeding shorebirds by blocking natural processes such as overwash that help to create and maintain high-quality beach habitat (Schupp et al. 2013; Gieder 2015) or by increasing erosion of salt marshes (Burdick et al. 2025). Only one study to date appears to have examined the interacting effects of SLR and ESL events on shorebirds (Seavey et al. 2011); however, the potential for serious impacts to coastal nesting species suggests that additional research is warranted.

### Impacts of ESL events on foraging and roosting shorebirds

Similar to the impacts of long-term SLR, ESL events may limit both intertidal foraging time and preferred roosting habitat for shorebirds. For species that roost in low-elevation habitats susceptible to inundation (e.g., beaches, sandbars, offshore reefs), an increase in the magnitude and/or frequency of ESL events may decrease the availability of high-quality roost sites. The potential impacts of these changes in habitat availability may already be seen in shorebird responses to extreme high tides in the present day. In some areas, shorebirds are adopting effective strategies to cope with increased inundation—such as the use of floating seagrass rafts as alternative roosting habitat by shorebirds in northwest Mexico (Garcia-Walther et al. 2023). In other cases, however, inundation of roosting habitat has led to decreases in survival, as birds are forced into suboptimal habitat with higher disturbance or predation risk (Griffin et al. 2023) (Fig. 5). Alternatively, a lack of roosting habitat may cause shorebirds to spend more time engaged in flight, resulting in increases in energy expenditure (Mann et al. 2017). Paired with the concurrent limitation of foraging time by extreme tides (Churchwell et al. 2018), this suggests that shorebirds may experience increased difficulty meeting their energy demands (Fig. 4). This problem may be particularly severe during migration, potentially limiting migration success or resulting in further knock-on or carry-over effects with implications for shorebird reproduction (Newton 2004; English 2022).

## Priorities for future research

In examining both the topical and geographical distribution of studies concerning impacts of SLR on the world’s shorebirds, clear knowledge gaps have emerged across species, flyways, and stages of the annual cycle (Figs. 2, S1). To date, SLR impacts have primarily been examined during the breeding season (23/37 studies, 62.2%) and across the two Atlantic flyways (29/37, 78.4%). Comparatively little research has been conducted across the African and Asian flyways (4/37, 10.8%), to examine potential changes in stopover habitat (5/37, 13.5%), or to examine impacts of SLR on shorebird populations during the migratory period (3/37, 8.1%). Furthermore, most studies identified through our review examined impacts of SLR at a single site or region, with only two studies (5.4%) analyzing impacts across the entire annual cycle. In light of these limitations, we identify four major research gaps that may serve as priorities for future work in the area of SLR impacts to shorebirds.

### Expanding research on SLR impacts in the Global South

To fully understand the threat of SLR to shorebirds on a global scale, future research should prioritize the study of SLR impacts at key shorebird sites in the Global South. Of the 37 studies identified through our literature review, only five (13.5%) focused either wholly or partly on countries situated in the Global South (Mexico: Garcia-Walther et al. 2024; Brazil: Santos et al. 2025; Saudi Arabia: AlRashidi et al. 2012; China and multiple countries in Southeast Asia: Iwamura et al. 2013; Nicol et al. 2015), with no studies taking place in Africa. This is despite the fact that sites throughout the Global South play a critical role for many shorebird species throughout the annual cycle, particularly during the non-breeding period. In the Western Hemisphere, Global South countries contain over half (69/125, 55.2%) of all WHSRN (Western Hemisphere Shorebird Reserve Network) sites, including 28 sites of hemispheric and international importance for shorebirds (Table S4). Similarly, along the East Asian–Australasian Flyway, over one-third (58/151, 38.4%) of important shorebird sites are located in the Global South (EAAFP 2024). While it is possible that relevant studies written in other languages may have been missed by our review process, research on SLR impacts outside the Global North may also be limited by financial inequalities. Accurate predictions of geomorphological and/or ecological change in response to SLR require high-resolution spatial data (e.g., LiDAR-derived digital elevation models) and other resources such as local tide gauges and modelling software, the availability of which may be limited in the Global South (Garcia-Walther et al. 2024). Indeed, the impact of SLR on estuaries in general appears to be severely understudied along much of the South American and African coastlines (Costa et al. 2023), likely as a result of such data limitations. Given the growing threat of SLR, the reality of financial inequalities, and the reliance of migratory shorebirds on a network of sites spread across multiple countries and even hemispheres, sharing of resources among countries and organizations to facilitate research on SLR impacts in the Global South should be a future priority for established international partnerships.

### Accounting for potential shifts in the distribution of coastal wetlands

Future studies should make greater use of ‘dynamic’ models (i.e., those that account for shifts in habitat with future SLR) to gain a more accurate understanding of SLR impacts on coastal ecosystems. Accommodation space (i.e., the amount of space inland/upland of coastal wetlands that is available for habitat expansion) is a key factor affecting the ability of coastal ecosystems to adapt to SLR (Schuerch et al. 2018). While accommodation space may be limited on developed coastlines due to the presence of anthropogenic barriers (Pontee 2013), coastal wetlands may persist or even expand with SLR in regions absent of such barriers (Kirwan et al. 2010; Santos et al. 2025). As such, accounting for the effects of accommodation space and allowing for the possibility of inland habitat migration during modelling is vital to build an accurate picture of SLR impacts on coastal ecosystems used by shorebirds. Despite this, over one-third of studies identified through our review (14/37, 37.8%) employed a ‘static’ modelling approach that did not allow intertidal habitats to shift with SLR (Fig. 6, Table S3), clouding our understanding of potential SLR impacts at these sites. The importance of considering accommodation space and allowing for habitat shifts is demonstrated by the few papers that employed both static and dynamic approaches (Seavey et al. 2011; Sims et al. 2013; Klingbeil et al. 2021). In all three cases, static modelling projected universal declines in habitat extent, while dynamic modelling predicted increases in habitat for certain sites and scenarios. Dynamic models thus provide a more realistic picture of the range of possible SLR impacts. These models may also be used to investigate the potential response of coastal ecosystems to different coastal adaptation solutions, making them extremely valuable not only for understanding the threat of SLR but also for developing practical approaches to address such threats. Static inundation modelling is still a useful analytical tool in regions where high-resolution spatial data are not available (e.g., Garcia-Walther et al. 2024) or for quantifying impacts at the flyway scale where dynamic modelling of individual sites may be impractical (e.g., Iwamura et al. 2013). However, future studies should aim to use dynamic models whenever possible to improve our understanding of SLR impacts to coastal wetlands and associated species.

### Quantifying population-level impacts of SLR on shorebirds

Studies wishing to draw conclusions about the magnitude of SLR impacts on shorebirds must move beyond simple examinations of habitat change to quantify impacts at the population level. The majority (27/37, 73.0%) of studies identified through our review reported impacts of SLR on shorebird habitat. However, translation of these indirect impacts into direct effects on shorebird individuals or populations occurred in only one-third (9/27, 33.3%) of cases. Predicting direct impacts to shorebird populations is more challenging due to a general lack of demographic data (Méndez et al. 2018) and full-annual-cycle population models (Hostetler et al. 2015) for most migratory species, leading many studies to draw conclusions about the impact of SLR on shorebirds simply by looking at changes in their habitat. However, such changes do not translate linearly into impacts on populations. Among the few studies that predicted changes to both shorebird habitat and populations, impacts to populations were typically more severe than expected based on simple declines in habitat extent (Aiello-Lammens et al. 2011; Convertino et al. 2011; Iwamura et al. 2013; Linhoss et al. 2013; van de Pol et al. 2024). This is likely due at least partially to migratory connectivity, which may cause populations to be disproportionately impacted by the degree of habitat change at a few key ‘bottleneck’ sites (Iwamura et al. 2013). Impacts to shorebird populations may also occur before habitat is lost, with SLR predicted to lead to increased rates of nest flooding (van de Pol et al. 2024), shifts in benthic invertebrate communities (Fujii 2006), and decreases in foraging time (Nightingale et al. 2018) (Fig. 4). Even eventual increases in the extent of foraging habitat may not necessarily benefit shorebirds, if areas of newly created habitat do not host the same abundance of food resources (Santos et al. 2025). Given these complexities, future research should aim to link projected changes in habitat extent and quality to demographic rates and population trajectories. Depending on the ecological mechanism, research question, and available data, this could involve demographic or stage-structured population models, integrated population models, full-annual-cycle models, or more data-intensive individual-based models (Hostetler et al. 2015).

### Considering trade-offs and the ability of shorebirds to adapt to future change

To aid in estimating population-level impacts, future research should more thoroughly examine the adaptive capacity of shorebirds and the extent to which behavioural adaptation may help to mitigate SLR impacts. The vast majority of studies included in our review did not account for the ability of shorebirds to adapt to SLR (e.g., through shifts in nest elevation, use of alternative foraging habitats, etc.). While our knowledge about the capacity of shorebirds to adapt to future change is limited—and adaptation ability may be highly variable depending on the species, location, and scenarios being examined—accounting for the possibility of behavioural shifts can lessen the magnitude of projected SLR impacts on shorebird populations (van de Pol et al. 2024). As such, the fact that most studies did not incorporate behavioural adaptation suggests that impacts of SLR on shorebirds may be less severe than predicted. However, any changes in shorebird behaviour to adapt to rising sea levels will inevitably involve trade-offs. For example, nesting at higher elevations may help to limit flood risk (Bailey et al. 2019) while simultaneously increasing predation risk and limiting the ability for parents and chicks to forage (van de Pol et al. 2010; Plaschke et al. 2019) (Fig. 4). In some cases, behavioural adjustments may also be insufficient to fully deal with a threat (Bailey et al. 2019; van de Pol et al. 2024). Thus, while the possibility of behavioural shifts should be accounted for, we should not assume that these changes will be sufficient to fully mitigate the impacts of future SLR, particularly for long-distance migrants that are exposed to multiple stressors across the annual cycle. Although the complexity of individual behaviour and uncertainty in the impacts of future climate change make successful adaptation pathways difficult to predict, gaining a better understanding of possible adaptations and their trade-offs—and incorporating these factors in future analyses—will help us to gain a more accurate picture of the extent to which shorebirds may be impacted by future SLR. Until these impacts are better understood, maintaining intact networks of high-quality sites will be vital to maximize the ability of shorebird populations to respond and adapt to future stressors (Martin et al. 2007; Bowgen et al. 2022).

## Conclusions

Evidence to date suggests that future SLR will impact coastal ecosystems and shorebird populations worldwide. Although considerable knowledge gaps remain concerning the severity of population-level impacts and the ability of shorebirds and their habitats to respond to future change, our findings suggest that SLR has important implications for shorebirds during all stages of the annual cycle, with declines in coastal wetland habitat and shorebird populations predicted in many regions of the globe. In light of this growing body of evidence, additional research is required not only to better understand the impacts of SLR on shorebirds but also to further develop and test the effectiveness of management interventions to address this threat. Given the importance of accommodation space for the maintenance of coastal wetlands, actions that will minimize the threat of coastal squeeze appear to provide a promising avenue for maximizing the ability of shorebirds to adapt to SLR (Iwamura et al. 2014). Potential strategies include the removal or alteration of artificial barriers (e.g., managed coastal realignment projects in the UK; NCCARF 2017), protection or restoration of existing upland to allow for natural expansion of intertidal habitats (e.g., inclusion of estuarine–upland transition zones in conservation planning for the San Francisco Estuary, USA; SFEP 2022), and enhancement of sediment supply to coastal wetlands (e.g., the Sturgeon Bank Sediment Enhancement Pilot Project in the Fraser River Estuary, Canada (DUC 2025); the Sand Engine in the Netherlands (Rijkswaterstaat 2025). This research should also be paired with studies examining the suitability of any newly created habitat for shorebirds of conservation concern (Santos et al. 2025). Ultimately, safeguarding migratory shorebirds from the impacts of SLR will require bold action and leadership to address the conflict between protecting human infrastructure from coastal flooding (e.g., through the continued construction and expansion of barriers that exacerbate coastal squeeze) and facilitating habitat protection for shorebirds through strategies such as managed retreat, which would allow critical habitats like salt marshes and mudflats to migrate inland with SLR.

## Supplementary Information

Below is the link to the electronic supplementary material.

Supplementary Material 1

Supplementary Material 2

## Data Availability

All data and code used for this study are publicly available through Zenodo (10.5281/zenodo.21462183).
