# Supplementary material for: Impacts of sea level rise on the world’s shorebirds
Source: Biodivers Conserv. 2026 Aug 10;35(10):236. doi: 10.1007/s10531-026-03431-8 (PMC13457410; doi:10.1007/s10531-026-03431-8)
Supplement: Supplementary file 1 [file 10531_2026_3431_MOESM1_ESM.pdf]

Biodiversity and Conservation

Supplementary materials

Impacts of sea level rise on the world's shorebirds

Jennifer M.T. Magel[1*], Scott Wilson[1,2*], and Tara G. Martin[1]

[1] Department of Forest & Conservation Sciences, University of British Columbia, Vancouver, BC, Canada V6T 1Z4

[2] Wildlife Research Division, Environment and Climate Change Canada, University of Alberta, Edmonton, AB, Canada T6G 2E9

*Corresponding authors: Jennifer M.T. Magel (jenn.magel@gmail.com)
Scott Wilson (ScottD.Wilson@ec.gc.ca)

Contents:

Supplementary methods
Figure S1
Tables S1–S4

Supplementary methods

Systematic literature review

Literature search

To ensure representation of a wide variety of sources—including journal articles, reports, theses/dissertations, and other forms of grey literature—we conducted a systematic literature search using three database search engines: Web of Science, Scopus, and ProQuest. The Web of Science Core Collection was accessed through a subscription provided by the University of British Columbia, which included access to the following indexes: Science Citation Index Expanded (1900–present), Social Sciences Citation Index (1956–present), Arts and Humanities Citation Index (1975–present), Conference Proceedings Citation Index-Science (1990–present), Conference Proceedings Citation Index-Social Sciences & Humanities (1990–present), and Emerging Sources Citation Index (2015–present). Scopus searches included all available databases, while the ProQuest search was limited to three databases (Agricultural & Environmental Science Collection; Earth, Atmospheric & Aquatic Science Collection; ProQuest Dissertations & Theses Global) to limit the number of irrelevant search results. Database searches were initially performed between 04 September and 07 October 2024 and were updated on 09 July 2026. All searches were conducted using the following search string: (shorebird* OR wader*) AND ("sea level rise" OR "sea-level rise"). However, search settings varied depending on the conventions of each individual search engine. For the Web of Science search, we limited the search terms to the 'topic' field (which searches the title, abstract, keyword plus, and author keywords for each paper). For the ProQuest search, we limited search terms to the 'abstract and summary text,' while for the Scopus search, we searched for the above terms in the 'article title, abstract, and keywords.' We did not implement any additional search restrictions (e.g., date range, language, document type, etc.).

Our search of the above three databases returned 187 unique results, which were systematically evaluated for inclusion in the review. In addition to those sources located through the systematic literature search, we included a further three studies previously located through ad hoc searches that were known to include data on SLR impacts to shorebirds (Durell et al. 2007; Gieder 2015; Klingbeil et al. 2021). The failure of our literature search to locate these three studies suggests several minor limitations in the review process. Firstly, our literature search was unable to locate relevant papers that were not indexed in at least one of the three databases, either because the journal in which the paper was published was not indexed or because the database was missing the volume from the year in which the paper was published (e.g., Durell et al. 2007). However, we expect such omissions to be relatively rare due to our use of multiple databases for the literature search. Secondly, we were unable to locate theses and dissertations that were not available through ProQuest (e.g., Gieder 2015). While such studies may be publicly available through university databases, searching the collections of individual universities would not be feasible for the purposes of this study. Finally, our search was unable to locate studies that did not refer to either 'shorebirds' or 'waders' in the title, abstract, or keywords, because shorebirds were not the main focus of the paper (e.g., Klingbeil et al. 2021). We acknowledge that our literature search may have failed to capture all available information

about SLR impacts to shorebirds if such information was contained within a study that focused on impacts to coastal waterbirds more broadly; however, expanding our literature search to include all papers related to SLR impacts on coastal birds for the purpose of locating such studies would also be infeasible for the purposes of our review.

Study screening

During each phase of the screening process (Fig. 1), papers were systematically evaluated by a single reviewer (JM). During the first phase of screening, we evaluated the title and abstract of each paper (n = 190) to determine whether the study documented impacts of SLR on shorebirds. In cases where this was difficult to discern (e.g., when it was unclear whether the paper actually quantified SLR impacts or such impacts were merely mentioned in the abstract as a possible implication of the results), studies were generally advanced to the next round of evaluation to be examined in more detail. A total of 94 studies were excluded from further consideration during this phase, as they either did not examine impacts of SLR (n = 82), did not examine impacts to shorebirds (n = 25), or did not include original data (n = 2; inclusion of original data was only assessed during the first phase of screening for very short publications with no abstract) (note that several studies fell under multiple exclusion criteria, so numbers do not add up to 94). Following the first phase of screening, a subset of 96 papers was advanced to the retrieval phase. We were unable to access the full texts for three of these papers, such that a subset of 93 papers was advanced to the final phase of screening.

During the final phase, we read through the full text of each study to determine whether it provided original quantitative data examining the impact of SLR on some aspect of shorebird ecology (e.g., habitat, food sources) or shorebird individuals/populations. Although the response of shorebirds to future SLR may be foreshadowed by the impacts of present-day phenomena such as extreme sea level (ESL) events resulting from king tides, storm surges, and hurricanes, we excluded studies focusing on ESL events from our review unless they explicitly used such data as a proxy to study SLR impacts (e.g., Nightingale et al. 2018). In cases where multiple studies were based around the same/similar datasets, we included all studies in the review as long as each study produced at least one novel result concerning SLR impacts to shorebirds. A total of 56 studies were excluded from further consideration during this phase, as they either did not examine SLR impacts (n = 30), did not produce original data (n = 17), did not include quantitative data (n = 4), did not examine shorebirds (n = 4), did not examine SLR impacts to shorebirds (n = 2; i.e., study discussed both shorebirds and SLR impacts but did not explicitly examine impacts of SLR on shorebirds), or did not isolate impacts of SLR from those of other stressors (n = 1).

Data extraction

For the 37 studies that met the eligibility criteria for inclusion in our review, the following data were extracted: author(s), year of publication, timeline, study length, country, region/site, flyway, species, stage of the annual cycle, type of impact examined, habitat response, and direction of impact.

Year of publication: We took the year of publication for each study to be the earliest date at which access to the final version of the study was provided to the public by the publisher, either in digital or

print format (e.g., for a study that was available online in 2016 prior to being published in print in 2017, we took the year of publication to be 2016).

Timeline and study length: We extracted the start and end year for each study from the text; in most cases, this corresponded to the start and end dates for SLR simulations (except for Laursen et al. 2023 and Santos et al. 2025, which examined past SLR). In cases where the baseline data for the study came from surveys conducted in multiple years, the latest year was taken as the start date (e.g., if baseline data were provided for 2014 and 2015, the start year was assumed to be 2015). In cases where the end year for the study was not specified due to the use of decadal SLR projections (i.e., projections averaged across an entire decade), the endpoint of the study was considered to be the mid-point of the decade in question (e.g., 2085 for projections extending through 'the 2080s' or ending in '2080–2089'). Study length was then calculated as the number of years between the start and end dates of the study.

Flyway: We assigned each study to one or more of the eight shorebird flyways (Pacific Americas, Mississippi Americas, Atlantic Americas, East Atlantic, Black Sea–Mediterranean, West Asian–East African, Central Asian, East Asian–Australasian) based on the location of the study site(s) as given in the text, the migratory route(s) of the species studied, and the flyway map provided in Boere and Stroud (2006). Site location was used for flyway assignments even in cases where the focal species was non-migratory, as individual sites may host multiple shorebird species. In cases where the focal species was resident at the study site and the site could potentially fall under more than one flyway, the site was assigned to both flyways. This was the case for one study (AlRashidi et al. 2012) that took place in the Farasan Islands, Saudi Arabia, which falls within both the Black Sea–Mediterranean and the West Asian–East African flyways.

Stage of the annual cycle: Studies were classified as 'breeding,' 'migration,' 'non-breeding' (or some combination thereof), 'resident,' 'year-round,' or 'full annual cycle' depending on the spatial scope of the study and the role of the study site(s) for the species being examined. In most cases, this was clear from the text. In cases where the stage of the annual cycle was not specified, we attempted to discern the appropriate stage using data about the species' migratory patterns from Birds of the World (Billerman et al. 2022) and eBird (Fink et al. 2024). This was necessary for two studies that simply purported to examine "foraging habitat" for several shorebird species along the coast of Georgia in the United States (Hunter et al. 2015) and in the German sector of the Wadden Sea (Laursen et al. 2023). The five foraging shorebird species in Hunter et al. (2015) (Piping plover, Red knot, Ruddy turnstone, Sanderling, Whimbrel) were classified as 'non-breeding' based on the range maps provided in Birds of the World. However, it should be noted that the Georgia coast may also serve as a stopover area for migrating individuals from some of these species (e.g., Red knot, Whimbrel) that primarily winter further south. The seven shorebird species in Laursen et al. (2023) were found to use the Wadden Sea during all stages of the annual cycle, with one species (Pied avocet) breeding in the region, three species (Bar-tailed godwit, Black-bellied plover, Dunlin) passing through during

migration, and three species (Common redshank, Eurasian curlew, Eurasian oystercatcher) found in substantial numbers in the area year-round (Billerman et al. 2022; Fink et al. 2024).

In cases where the species of concern was resident to the area but the study specifically examined a factor related to the breeding period, the study was classified as 'breeding.' This was the case for three studies (Convertino et al. 2011; AlRashidi et al. 2012; Convertino et al. 2012) that focused on nesting habitat for Snowy plover and Kentish plover in areas where the species are found year-round. In cases where the species of concern was non-migratory and the study did not specifically look at nesting habitat, the study was labelled as 'resident.' This was the case for only one study (Hollenbeck et al. 2014), which focused on Black oystercatcher along the coast of Oregon in the United States. Studies were classified as 'year-round' if they focused on migratory species, they did not specifically examine impacts to breeding/nesting habitat, and at least one of the species examined was present at the site year-round. This was the case for one study (Laursen et al. 2023, described above). Finally, studies were classified as 'full annual cycle' if they examined impacts of SLR across the entire flyway. This was the case for two studies (Iwamura et al. 2013; Nicol et al. 2015) that quantified impacts of SLR on habitat and shorebird populations across multiple countries of the East Asian–Australasian Flyway.

<u>Species and family:</u> We considered shorebirds to be any of the ~215 species within the following 14 families in the Order Charadriiformes, in accordance with the taxonomic overview provided in Colwell (2010): Burhinidae, Charadriidae, Chionidae, Dromadidae, Glareolidae, Haematopodidae, Ibidorhynchidae, Jacanidae, Pedionomidae, Pluvianellidae, Recurvirostridae, Rostratulidae, Scolopacidae, and Thinocoridae. It should be noted that several of these families include species that spend the entire annual cycle on interior grasslands and plateaus (e.g., Ibidorhynchidae, Pedionomidae, Thinocoridae) and would, therefore, not be expected to be impacted by SLR. However, the number of species that spend at least part of the annual cycle in coastal ecosystems has—to the best of our knowledge—not been quantified. As such, we do not draw any conclusions about the proportion of coastal shorebird species for which SLR impacts have been quantified. Rather, we simply use this classification as a means of identifying studies concerning our species of interest and distinguishing them from studies focusing on other coastal waterbird species (e.g., terns, rails) that are sometimes referred to as 'shorebirds.'

For each species, we use the most recently agreed upon scientific name and English name as reported in Birds of the World (Billerman et al. 2022). In some cases, these were different from the names used in the original study due to taxonomic revisions and subsequent changes in the English and/or scientific names (e.g., re-naming of the Lesser sand plover *Charadrius mongolus* to the Siberian sand plover *Anarhynchus mongolus*) or differences in English names between Europe and the Americas (e.g., *Pluvialis squatarola* is referred to herein as 'Black-bellied plover' rather than 'Grey plover'). Family names were similarly extracted from Birds of the World (Billerman et al. 2022). Standardized four-letter codes were assigned to each species for the purpose of plotting based on the conventions used by the Institute for Bird Populations (Pyle and DeSante 2024). For the two species—Kentish plover and Pied avocet—for which codes were not available, we assigned the

codes ‘KEPL’ and ‘PIAV,’ respectively, which are not currently used by any of the other species listed in Pyle and DeSante (2024).

Type of impact: Data on SLR impacts were extracted at two levels. First, studies were coarsely classified based on whether they quantified impacts of SLR on shorebird habitat vs. impacts to shorebird individuals or populations. Following this, we extracted data on the specific impact(s) examined by each study. Thirty metrics were identified in total: 13 impacts to shorebird habitat (changes in habitat extent, carrying capacity, habitat suitability, geomorphology, habitat extent threatened by storm flooding, habitat fragmentation, habitat patch size, habitat permanence, likelihood of overwash, prey biomass, prey density, probability of nest flooding, and vulnerability to erosion) and 17 impacts to shorebird individuals or populations (changes in overwinter survival or mortality, foraging time, site-level abundance, extinction risk, mean energy stores, percent decline in population, population flow, breeding population size, dietary composition, expected minimum abundance, habitat use, metapopulation size, population growth rate, population size, probability of decline to 50 individuals, risk of decline to 20 individuals, and site-level density).

Habitat response: Studies were classified as ‘static’ or ‘dynamic’ depending on whether they accounted for potential shifts in the distribution of intertidal habitat with future SLR. Studies that employed ‘bathtub’ inundation models or that otherwise modelled SLR by shifting the lower elevational boundary of intertidal habitat patches (e.g., studies that looked at changes in patch height) were classified as ‘static.’ Conversely, studies that employed models such as SLAMM (the Sea Level Affecting Marshes Model) or that otherwise allowed for the possibility of habitat expansion with future SLR (e.g., studies that examined changes in shorebird survival given both increases and decreases in patch size) were classified as ‘dynamic.’

Direction of impact: Data on the direction of SLR impacts (positive, negative, mixed) were extracted for each individual SLR scenario, site, species, and habitat/population metric examined in the study. Although many studies examined impacts of SLR at multiple timesteps, we extracted data only for the final timestep (i.e., the endpoint) of each study for consistency. We first assigned each study a ‘mean’ direction of impact by averaging results across sites, species, and/or scenarios within the same study (e.g., examining the mean change in habitat area or shorebird abundance across all scenarios for studies that examined multiple scenarios, or across all sites for studies that examined multiple sites). A value of ‘mixed’ was assigned here in cases where the direction of impact varied but it was not possible to calculate a mean value using the given data (e.g., if data was presented only in a low-resolution figure and could not reliably be extracted). We then assigned a direction of impact for each of the other factors (scenario, site, species, and habitat/population metric, as necessary) by calculating the mean direction of impact for each level of the factor in question, averaged across all other factors (e.g., calculating the net change in habitat area or bird abundance for each SLR scenario, averaged across sites and/or species). Here, a value of ‘positive’ or ‘negative’ was assigned only in cases where the direction of impact was uniform across all levels of the factor in question. For example, a direction of ‘negative’ was assigned for ‘scenario’ if the mean direction of impact was

negative (e.g., a net decrease in habitat area was observed) across all scenarios, while a value of 'mixed' was assigned if the mean direction of impact varied across scenarios (e.g., if a net decrease in habitat area was predicted for two scenarios and a net increase predicted for the third scenario). For studies that examined multiple habitat or population metrics (e.g., habitat extent and prey availability; shorebird density and abundance), a value of 'negative' or 'positive' was assigned if the direction of change was the same for both metrics, while a value of 'mixed' was assigned if the direction of change varied (e.g., if the study predicted decreases in density but increases in abundance).

References

AlRashidi, M., Shobrak, M., Al-Eissa, M.S., and Székely, T. 2012. Integrating spatial data and shorebird nesting locations to predict the potential future impact of global warming on coastal habitats: A case study on Farasan Islands, Saudi Arabia. Saudi J Biol Sci **19**(3): 311–315. doi:10.1016/j.sjbs.2012.02.001.

Billerman, S.M., Keeney, B.K., Rodewald, P.G., and Schulenberg, T.S. (*Editors*). 2022. Birds of the World. Cornell Lab of Ornithology, Ithaca, NY, USA. Available from https://birdsoftheworld.org/bow/home.

Boere, G.C., and Stroud, D.A. 2006. The flyway concept: what is it and what it isn't. *In* Waterbirds around the world. *Edited by* G.C. Boere, C.A. Galbraith, and D.A. Stroud. The Stationery Office, Edinburgh, UK. pp. 40–47.

Colwell, M.A. 2010. Shorebird Ecology, Conservation, and Management. University of California Press, Los Angeles, USA.

Convertino, M., Bockelie, A., Kiker, G.A., Muñoz-Carpena, R., and Linkov, I. 2012. Shorebird patches as fingerprints of fractal coastline fluctuations due to climate change. Ecol Process **1**(1): 9. doi:10.1186/2192-1709-1-9.

Convertino, M., Kiker, G.A., Chu-Agor, M.L., Muñoz-Carpena, R., Martinez, C.J., Aiello-Lammens, M., Akçakaya, H.R., Fischer, R.A., and Linkov, I. 2011. Integrated modeling to mitigate climate change risk due to sea level rise: imperiled shorebirds on Florida coastal military installations. *In* Climate: Global Change and Local Adaptation. *Edited by* I. Linkov and T.S. Bridges. Springer, Dordrecht, The Netherlands. pp. 433–464.

Durell, S.E.A. le V. dit, Stillman, R.A., McGrorty, S., West, A.D., and Price, D.J. 2007. Predicting the effect of local and global environmental change on shorebirds: a case study on the Exe estuary, UK. Wader Study Group Bull **112**: 24–36.

Fink, D., Auer, T., Johnston, A., Strimas-Mackey, M., Ligocki, S., Robinson, O., Hochachka, W., Jaromczyk, L., Crowley, C., Dunham, K., Stillman, A., Davis, C., Stokowski, M., Sharma, P., Pantoja, V., Burgin, D., Crowe, P., Bell, M., Ray, S., Davies, I., Ruiz-Gutierrez, V., Wood, C., and Rodewald, A. 2024. eBird Status and Trends, Data Version: 2023; Released: 2025. Cornell Lab of Ornithology, Ithaca, USA. doi:https://doi.org/10.2173/WZTW8903.

Gieder, K.D. 2015. Assessing the effects of sea-level rise on Piping plover (*Charadrius melodus*) nesting habitat, and the ecology of a key mammalian shorebird predator, on Assateague Island. PhD thesis, Virginia Polytechnic Institute and State University, Blacksburg, USA.

Hunter, E.A., Nibbelink, N.P., Alexander, C.R., Barrett, K., Mengak, L.F., Guy, R.K., Moore, C.T., and Cooper, R.J. 2015. Coastal vertebrate exposure to predicted habitat changes due to sea level rise. Environ Manage **56**(6): 1528–1537. doi:10.1007/s00267-015-0580-3.

Iwamura, T., Possingham, H.P., Chadès, I., Minton, C., Murray, N.J., Rogers, D.I., Treml, E.A., and Fuller, R.A. 2013. Migratory connectivity magnifies the consequences of habitat loss from sea-level rise for shorebird populations. Proc R Soc B Biol Sci **280**(1761): 20130325. doi:10.1098/rspb.2013.0325.

Klingbeil, B.T., Cohen, J.B., Correll, M.D., Field, C.R., Hodgman, T.P., Kovach, A.I., Lentz, E.E., Olsen, B.J., Shriver, W.G., Wiest, W.A., and Elphick, C.S. 2021. High uncertainty over the future of tidal marsh birds under current sea-level rise projections. Biodivers Conserv **30**(2): 431–443. doi:10.1007/s10531-020-02098-z.

Laursen, K., Bregnballe, T., Kleefstra, R., Frikke, J., Guenther, K., Hornman, M., Pedersen, C.L., Blew, J., and Pape Moller, A. 2023. Regime shift and changes in sediment morphology driven by sea level rise affect abundance of migratory waders. J Ornithol **164**(3): 573–582. doi:10.1007/s10336-023-02056-w.

Nicol, S., Fuller, R.A., Iwamura, T., and Chadès, I. 2015. Adapting environmental management to uncertain but inevitable change. Proc R Soc B Biol Sci **282**(1808): 20142984. doi:10.1098/rspb.2014.2984.

Nightingale, J., Stevenson, D., and Rowsell, E. 2018. Activity budgets of a wintering population of Purple Sandpipers: Vulnerability to sea-level rise and conservation through coastal engineering? Wader Study **125**(2): 122–128.

Pyle, P., and DeSante, D.F. 2024. Four-letter (English name) and six-letter (scientific name) alpha codes for 2022 bird species (and 139 non-species taxa) in accordance with the 65th AOS Supplement (2024), sorted alphabetically by English name. The Institute for Bird Populations, Petaluma, USA. Available from https://www.birdpop.org/docs/misc/Alpha_codes_eng.pdf.

Santos, C.D., Paludo, D., Silvestro, P., Monteiro, J.O., Pachelle, P.P.G., Oliveira, V., and Mobley, J.A. 2025. Sea-level rise causes feeding habitat loss for migratory shorebirds in remote coastal wetlands of Brazilian Amazon. Environ Res Lett **20**(3): 034038. doi:10.1088/1748-9326/adb444.

WHSRN. 2019. WHSRN sites. Available from https://whsrn.org/whsrn-sites/.

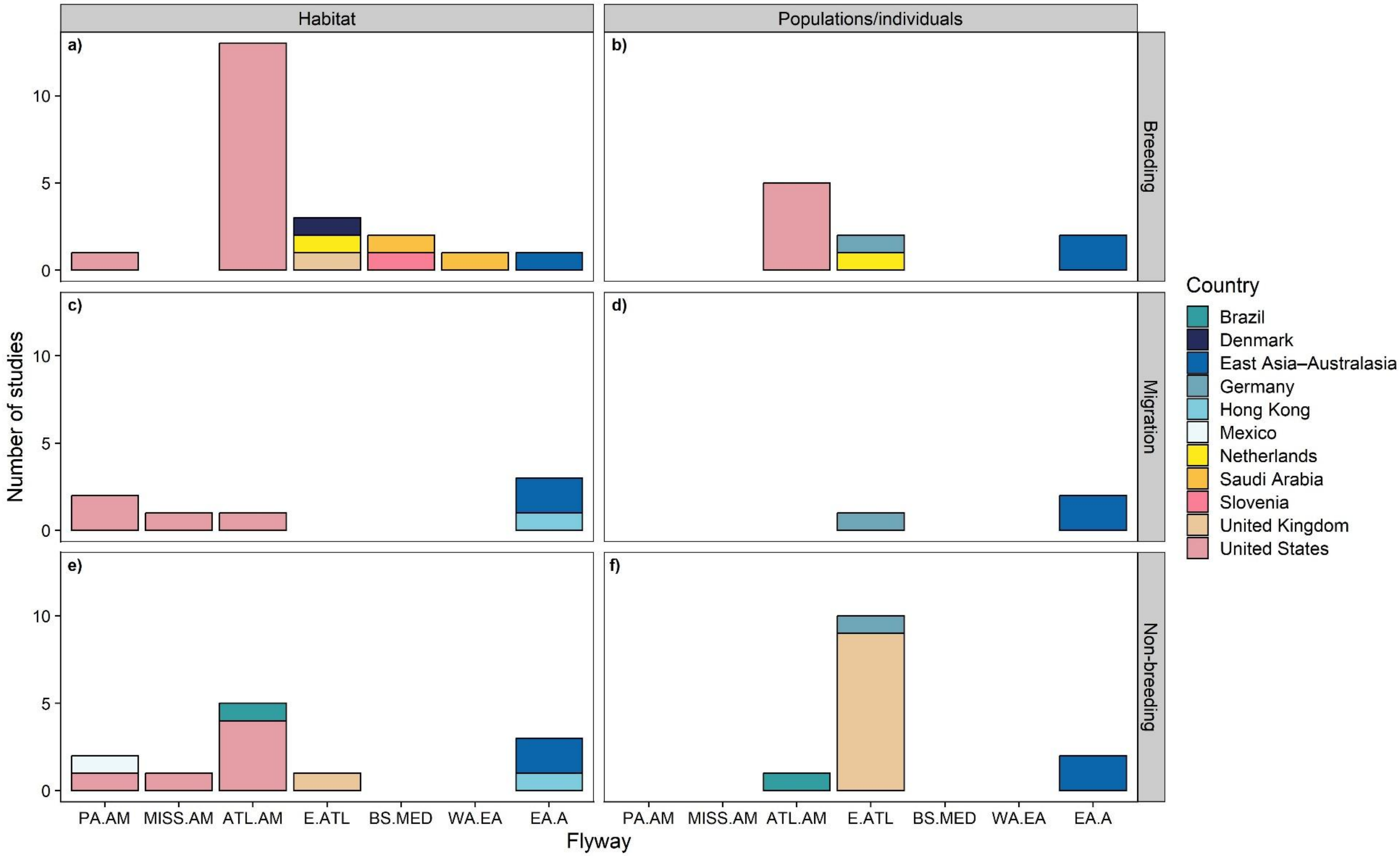

Figure S1. Number of studies published to date examining impacts of sea level rise on the world's shorebirds across countries and flyways, divided by the stage of the annual cycle (top = breeding; middle = migration; bottom = non-breeding) and type of impact examined (left = habitat; right = shorebird populations/individuals). Studies examining multiple metrics or stages of the annual cycle are represented in all relevant panels. PA.AM = Pacific Americas, MISS.AM = Mississippi Americas, ATL.AM = Atlantic Americas, E.ATL = East Atlantic, BS.MED = Black Sea–Mediterranean, WA.EA = West Asian–East African, and EA.A = East Asian–Australasian.

Table S1. Studies (n = 37) identified through the systematic literature review that have examined impacts of past or future sea level rise on shorebirds. Studies are divided based on whether they examined impacts to shorebird habitat vs. individuals/populations and are further subdivided based on the stage of the annual cycle examined. Within each subdivision, studies are arranged in reverse chronological order, starting with the most recent. See below for a list of codes corresponding to flyway abbreviations; see Table S2 for common and scientific names corresponding to each species code.

**Author & year**	**Title**	**Timeline**	**Flyway**	**Site (country)**	**Species**	**Type of impact**	
						**Coarse**	**Fine**
Studies examining impacts of SLR on shorebird habitat							
Breeding							
Harmer 2023	Determinants of nesting and nest success in saltmarsh breeding Common redshank, *Tringa totanus*, in North West England	2017–2100	E.ATL	Ribble estuary (UK)	CREH	Habitat	Habitat extent
Zeigler et al. 2022	Predicted sea-level rise-driven biogeomorphological changes on Fire Island, New York: implications for people and plovers	2015–2050	ATL.AM	Fire Island (USA)	PIPL	Habitat	Habitat extent; geomorphology; probability of overwash
McCarthy 2022	Planning for a disrupted future: modeling the effects of sea level rise on endangered shorebird habitat on Nantucket Island	2016–2100	ATL.AM	Nantucket Island (USA)	AMOY PIPL	Habitat	Habitat extent
Ivajnšič et al. 2016	The sea level rise impact on four seashore breeding birds: the key study of Sečovlje Salina Nature Park	2010–2100	BS.MED	Sečovlje Salina Nature Park (Slovenia)	KEPL	Habitat	Habitat extent
Gieder 2015	Assessing the effects of sea-level rise on Piping plover (*Charadrius melodus*) nesting habitat, and the ecology of a key mammalian predator, on Assateague Island	2008–2100	ATL.AM	Assateague Island (USA)	PIPL	Habitat	Habitat extent; habitat suitability
Sims et al. 2013	Room to move? Threatened shorebird habitat in the path of sea level rise—dynamic beaches, multiple users, and mixed ownership: a case study from Rhode Island, USA	2011–2100	ATL.AM	Rhode Island (USA)	PIPL	Habitat	Habitat extent

Clausen et al. 2013	Grazing management can counteract the impacts of climate change-induced sea level rise on salt marsh-dependent waterbirds	2007(?)–2095	E.ATL	Wadden Sea, Limfjord, Baltic Sea (Denmark)	BLTG, DUNL, RUFF	Habitat	Habitat extent
AlRashidi et al. 2012	Integrating spatial data and shorebird nesting locations to predict the potential future impact of global warming on coastal habitats: a case study on Farasan Islands, Saudi Arabia	n/a (1 m SLR)	BS.MED WA.EA	Farasan Islands (Saudi Arabia)	KEPL	Habitat	Habitat extent
Seavey et al. 2011	Effect of sea-level rise on piping plover (*Charadrius melodus*) breeding habitat	2005–2095(?)	ATL.AM	Long Island (USA)	PIPL	Habitat	Habitat extent; habitat extent threatened by storm flooding
Chu-Agor et al. 2011	Exploring vulnerability of coastal habitats to sea level rise through global sensitivity and uncertainty analyses	2001–2100	ATL.AM	Elgin Air Force Base, Florida (USA)	PIPL, SNPL	Habitat	Habitat extent
Non-breeding							
Garcia-Walther et al. 2024	Future sea-level rise in northwest Mexico is projected to decrease the distribution and habitat quality of the endangered *Calidris canutus roselaari* (Red Knot)	2016–2050	PA.AM	Northwest Mexico	REKN	Habitat	Habitat extent; habitat suitability
Breeding and migration							
Naughton 2013	SLAMM (Sea Level Affecting Marshes Model) modeling of the effects of sea level rise on coastal wetland habitat of San Diego County	2002–2100	PA.AM	San Diego County, San Diego Bay, Tijuana estuary (USA)	n/a	Habitat	Habitat extent
Breeding and non-breeding							
Von Holle et al. 2019	Effects of future sea level rise on coastal habitat	2000–2030	ATL.AM	South Atlantic Bight (USA)	AMOY, PIPL, WIPL	Habitat	Vulnerability to erosion
Hunter et al. 2015	Coastal vertebrate exposure to predicted habitat changes due to sea level rise	2007–2100	ATL.AM	Georgia (USA)	AMOY, PIPL, REKN, RUTU, SAND, WHIM, WIPL	Habitat	Habitat extent; habitat patch size; habitat permanence
Convertino et al. 2012	Shorebird patches as fingerprints of fractal coastline fluctuations due to climate change	2006–2100	ATL.AM	Florida Gulf Coast (USA)	PIPL, REKN, SNPL	Habitat	Habitat extent; habitat fragmentation

Migration and non-breeding							
Wikramanayake et al. 2020	A climate adaptation strategy for Mai Po Inner Deep Bay Ramsar site: stepping stone to climate proofing the East Asian–Australasian Flyway	2019–2100	EA.A	Mai Po Inner Deep Bay (Hong Kong)	n/a	Habitat	Habitat extent
Galbraith et al. 2002	Global climate change and sea level rise: potential losses of intertidal habitat for shorebirds	1990(?)–2100	PA.AM<br><br><br><br>MISS.AM<br>ATL.AM	Willapa Bay, Humboldt Bay, San Francisco Bay<br>Bolivar Flats<br>Delaware Bay (USA)	n/a	Habitat	Habitat extent
Non-migratory (resident) species							
Hollenbeck et al. 2014	Using terrestrial laser scanning to support ecological research in the rocky intertidal zone	2011–2100	PA.AM	Rabbit Rock, Oregon (USA)	BLOY	Habitat	Habitat extent
Studies examining impacts of SLR on shorebird populations/individuals							
Breeding							
Aiello-Lammens and Akçakaya 2017	Using global sensitivity analysis of demographic models for ecological impact assessment	2010–2100	ATL.AM	Florida Gulf Coast (USA)	SNPL	Bird	Probability of decline to 50 individuals
Non-breeding							
Nightingale et al. 2018	Activity budgets of a wintering population of non-breeding Purple Sandpipers: vulnerability to sea level rise and conservation through coastal engineering?	2017–2100	E.ATL	Southsea Castle, Portsmouth (UK)	PUSA	Bird	Foraging time
Collop 2017	Impact of human disturbance on coastal birds: population consequences derived from behavioural responses	2010–2060	E.ATL	Poole Harbour (UK)	BBPL, BLTG, BTGO, CREH, DUNL, EUCU, EUOY	Bird	Survival; foraging time; energy stores
Bowgen 2017	Predicting the effect of environmental change on wading birds: insights from individual-based models	2012–2095	E.ATL	Exe, Humber, and Severn estuaries; Southampton Water; Poole Harbour (UK)	BBPL, BLTG, BTGO, CREH, CRPL, DUNL, EUCU, EUOY, REKN, RUTU, SAND	Bird	Survival; dietary composition; habitat use

Ross 2013	Investigating the physical and ecological drivers of change in a coastal ecosystem: from individual- to population-scale impacts	2000–2095	E.ATL	Poole Harbour (UK)	PIAV	Bird	Survival; energy stores
Durell et al. 2007	Predicting the effects of local and global environmental change on shorebirds: a case study on the Exe estuary, U.K.	n/a (0–50 cm SLR)	E.ATL	Exe estuary (UK)	BBPL, BLTG, BTGO, DUNL, EUCU, EUOY	Bird	Survival
Durell et al. 2006	Modelling the effect of environmental change on shorebirds: A case study on Poole Harbour, UK	n/a (0–50 cm SLR)	E.ATL	Poole Harbour (UK)	BLTG, CREH, DUNL, EUCU, EUOY	Bird	Survival
Stillman et al. 2005	Predicting site quality for shorebird communities: a case study on the Humber estuary, UK	n/a (altered habitat area)	E.ATL	Humber estuary (UK)	BBPL, BLTG, BTGO, CREH, CRPL, DUNL, EUCU, EUOY, REKN	Bird	Survival
Austin and Rehfisch 2003	The likely impact of sea level rise on waders (Charadrii) wintering on estuaries	1998(?)–2055(?)	E.ATL	Debben and Duddon estuaries (UK)	CREH, DUNL, EUCU, EUOY, REKN	Bird	Abundance; density
Year-round							
Laursen et al. 2023	Regime shift and changes in sediment morphology driven by sea level rise affect abundance of migratory waders	1987–2019	E.ATL	Wadden Sea (Germany)	BBPL, BTGO, CREH, DUNL, EUCU, EUOY, PIAV	Bird	Abundance
Studies examining impacts of SLR on both shorebird habitat and populations/individuals							
Breeding							
van de Pol et al. 2024	Sea-level rise causes shorebird population collapse before habitats drown	1986–2125	E.ATL	Wadden Sea (Netherlands)	EUOY	Habitat<br>Bird	Habitat extent; probability of nest flooding<br>Population growth rate; percent decline in population
Klingbeil et al. 2021	High uncertainty over the future of tidal marsh birds under current sea-level rise predictions	2010–2050	ATL.AM	Maine to Virginia (USA)	WILL	Habitat<br>Bird	Habitat extent<br>Population size

Linhoss et al. 2013	Decision analysis for species preservation under sea-level rise	2010–2100	ATL.AM	Florida Gulf Coast (USA)	SNPL	Habitat<br><br>Bird	Habitat extent; carrying capacity<br>Extinction risk; population decline
Aiello-Lammens et al. 2011	The impact of sea level rise on Snowy plovers in Florida: integrating geomorphological, habitat, and metapopulation models	2010–2100	ATL.AM	Florida Gulf Coast (USA)	SNPL	Habitat<br><br>Bird	Habitat extent; carrying capacity<br>Extinction risk; risk of decline to 20 individuals; expected minimum abundance
Convertino et al. 2011	Integrated modeling to mitigate climate change risk due to sea level rise: imperiled shorebirds on Florida coastal military installations	2010–2100	ATL.AM	Florida Gulf Coast (USA)	SNPL	Habitat<br><br>Bird	Habitat extent; carrying capacity<br>Metapopulation size
Non-breeding							
Santos et al. 2025	Sea-level rise causes feeding habitat loss for migratory shorebirds in remote coastal wetlands of Brazilian Amazon	1987–2018	ATL.AM	Amapá (Brazil)	SESA	Habitat<br><br>Bird	Habitat extent; prey density<br>Abundance
Fujii 2006	Impacts of sea-level rise on an estuarine ecosystem: a case study of the Humber estuary, UK	n/a (10–50 cm SLR)	E.ATL	Humber estuary (UK)	n/a (examined benthic-feeding shorebirds in general)	Habitat<br><br>Bird	Habitat extent; prey biomass<br>Abundance
Full annual cycle							
Nicol et al. 2015	Adapting environmental management to uncertain but inevitable change	n/a (1–2 m SLR)	EA.A	Multiple countries in East Asia–Australasia	BTGO, CUSA, FECU, GRKN, GTTA, REKN, SISP, TESA	Habitat<br>Bird	Habitat extent<br>Population flow; breeding population size
Iwamura et al. 2013	Migratory connectivity magnifies the consequences of habitat loss from sea-level rise for shorebird populations	n/a (0.5–3 m SLR)	EA.A	Multiple countries in East Asia–Australasia	BTGO, CUSA, FECU, GRKN, GTTA, REKN, SISP, TESA	Habitat<br>Bird	Habitat extent<br>Population flow

Flyways ATL.AM = Atlantic Americas Flyway; BS.MED = Black Sea–Mediterranean Flyway; E.ATL = East Atlantic Flyway; EA.A = East Asian–Australasian Flyway; MISS.AM = Mississippi Americas Flyway; PA.AM = Pacific Americas Flyway; WA.EA = West Asian–East African Flyway (see Boere and Stroud 2006 for additional information)

Table S2. Species that have been the focus of at least one study examining impacts of sea level rise on shorebirds, number of studies conducted for each species, and stages of the annual cycle during which studies took place (B = breeding; M = migration; NB = non-breeding; FAC = full annual cycle; YR = year-round).

Family	Common name	Scientific name	Code	Number of studies					
				B	M	NB	FAC	YR	Total
Charadriidae (7 spp.)	Black-bellied plover	*Pluvialis squatarola*	BBPL		1	4			5
	Common ringed plover	*Charadrius hiaticula*	CRPL			2			2
	Kentish plover	*Anarhynchus alexandrinus*	KEPL*	2					2
	Piping plover	*Charadrius melodus*	PIPL	6		3			9
	Siberian sand plover	*Anarhynchus mongolus*	SISP				2		2
	Snowy plover	*Anarhynchus nivosus*	SNPL	6					6
	Wilson's plover	*Anarhynchus wilsonia*	WIPL	2					2
Haemotopodidae (3 spp.)	American oystercatcher	*Haematopus palliatus*	AMOY	3					3
	Black oystercatcher	*Haematopus bachmani*	BLOY	-	-	-	-	-	1
	Eurasian oystercatcher	*Haematopus ostralegus*	EUOY	1		6		1	8
Recurvirostridae (1 sp.)	Pied avocet	*Recurvirostra avosetta*	PIAV*	1		1			2
Scolopacidae (18 spp.)	Bar-tailed godwit	*Limosa lapponica*	BTGO		1	4	2		7
	Black-tailed godwit	*Limosa limosa*	BLTG	1		5			6
	Common redshank	*Tringa totanus*	CREH	1		5		1	7
	Curlew sandpiper	*Calidris ferruginea*	CUSA				2		2
	Dunlin	*Calidris alpina*	DUNL	1	1	6			8
	Eurasian curlew	*Numenius arquata*	EUCU			6		1	7
	Far eastern curlew	*Numenius madagascariensis*	FECU				2		2
	Great knot	*Calidris tenuirostris*	GRKN				2		2
	Grey-tailed tattler	*Tringa brevipes*	GTTA				2		2
	Purple sandpiper	*Calidris maritima*	PUSA			1			1
	Red knot	*Calidris canutus*	REKN			6	2		8
	Ruddy turnstone	*Arenaria interpres*	RUTU			2			2
	Ruff	*Calidris pugnax*	RUFF	1					1
	Sanderling	*Calidris alba*	SAND			2			2
	Semipalmated sandpiper	*Calidris pusilla*	SESA			1			1
	Terek sandpiper	*Xenus cinereus*	TESA				2		2
	Whimbrel	*Numenius phaeopus*	WHIM			1			1
	Willet	*Tringa semipalmata*	WILL	1					1

* Species code assigned by the authors due to lack of a four-letter code in Pyle and DeSante (2024) | - species is non-migratory

Table S3. Direction of impact (positive, negative, mixed) for studies that have examined the impacts of future sea level rise on (a) shorebird habitat and (b) shorebird populations or individuals. For all factors (overall, scenario, site, species, metric), direction of impact was assigned based on the net change in shorebird habitat or populations for that particular factor, averaged across all other factors (e.g., a value of 'negative' for 'Scenario' means that the net change in shorebird habitat, abundance, etc. averaged across sites and/or species was negative for all scenarios examined; see Supplementary Methods for further details on classification). Studies are additionally classified based on whether they accounted for the possibility of shifts in habitat distribution with future sea level rise (static = no; dynamic = yes). Two studies examining impacts of past SLR (Laursen et al. 2023, Santos et al. 2025) were excluded from this table.

Author & year	Habitat response	Overall	Scenario	Site	Species	Metric
(a) Studies examining impacts of SLR on shorebird habitat						
Breeding						
van de Pol et al. 2024	dynamic	negative	negative	negative	n/a	n/a
Harmer 2023	static	negative	negative	n/a	n/a	n/a
Zeigler et al. 2022	dynamic	negative	negative	n/a	n/a	n/a
McCarthy 2022	dynamic	negative	negative	negative	n/a	n/a
Klingbeil et al. 2021	static	negative	n/a	negative	n/a	n/a
	dynamic	negative	n/a	negative	n/a	n/a
VonHolle et al. 2019	dynamic	negative	n/a	n/a	negative	negative
Ivajnšič et al. 2016	static	negative	negative	n/a	n/a	n/a
Hunter et al. 2015	dynamic	negative	n/a	n/a	negative	negative
Gieder 2015	dynamic	positive	positive	n/a	n/a	n/a
Sims et al. 2013	static	negative	negative	mixed	n/a	n/a
	dynamic	positive	positive	mixed	n/a	n/a
Naughton 2013	dynamic	negative	negative	mixed	n/a	n/a
Linhoss et al. 2013	dynamic	negative	mixed	n/a	n/a	negative
Iwamura et al. 2013	static	negative	negative	negative	negative	n/a
Clausen et al. 2013	static	negative	negative	negative	n/a	n/a
Convertino et al. 2012	dynamic	negative	n/a	n/a	n/a	negative
AlRashidi et al. 2012	static	negative	n/a	n/a	n/a	negative
Seavey et al. 2011	static	negative	negative	n/a	n/a	n/a
	dynamic	positive	positive	n/a	n/a	n/a
Convertino et al. 2011	dynamic	negative	negative	mixed	n/a	n/a
Chu-Agor et al. 2011	dynamic	negative	negative	n/a	n/a	n/a
Aiello-Lammens et al. 2011	dynamic	negative	negative	n/a	n/a	n/a
Migration						
Wikramanayake et al. 2020	dynamic	negative	negative	n/a	n/a	n/a
Nicol et al. 2015	static	negative	negative	negative	negative	n/a
Naughton 2013	dynamic	mixed	mixed	mixed	n/a	n/a
Iwamura et al. 2013	static	negative	negative	negative	negative	n/a
Galbraith et al. 2002	dynamic	negative	mixed	mixed	n/a	n/a

Non-breeding						
Garcia-Walther et al. 2024	static	negative	n/a	mixed	n/a	negative
Wikramanayake et al. 2020	dynamic	negative	negative	n/a	n/a	n/a
Von Holle et al. 2019	dynamic	negative	n/a	n/a	n/a	negative
Nicol et al. 2015	static	negative	negative	negative	negative	n/a
Hunter et al. 2015	dynamic	positive	n/a	n/a	mixed	positive
Iwamura et al. 2013	static	negative	negative	negative	negative	n/a
Convertino et al. 2012	dynamic	negative	n/a	n/a	negative	negative
Fujii 2006	static	negative	negative	n/a	n/a	negative
Galbraith et al. 2002	dynamic	negative	mixed	mixed	n/a	n/a
Non-migratory (resident)						
Hollenbeck et al. 2014	dynamic	negative	mixed	n/a	n/a	mixed
(b) Studies examining impacts of SLR on shorebird populations/individuals						
Breeding						
van de Pol et al. 2024	dynamic	negative	negative	negative	n/a	n/a
Klingbiel et al. 2021	static	negative	n/a	n/a	n/a	n/a
	dynamic	negative	n/a	n/a	n/a	n/a
Aiello-Lammens and Akçakaya 2017	dynamic	negative	n/a	n/a	n/a	n/a
Nicol et al. 2015	static	negative	negative	negative	negative	negative
Linhoss et al. 2013	dynamic	negative	negative	n/a	n/a	n/a
Iwamura et al. 2013	static	negative	negative	negative	negative	n/a
Aiello-Lammens et al. 2011	dynamic	negative	negative	n/a	n/a	negative
Convertino et al. 2011	dynamic	negative	negative	n/a	n/a	n/a
Migration						
Nicol et al. 2015	static	negative	negative	negative	negative	negative
Iwamura et al. 2013	static	negative	negative	negative	negative	n/a
Non-breeding						
Nightingale et al. 2018	static	negative	negative	n/a	n/a	n/a
Collop 2017	static	negative	negative	n/a	mixed	mixed
Bowgen 2017	static	negative	negative	mixed	mixed	n/a
Nicol et al. 2015	static	negative	negative	negative	negative	negative
Ross 2013	static	negative	negative	n/a	n/a	mixed
Iwamura et al. 2013	static	negative	negative	negative	negative	n/a
Durell et al. 2007	static	negative	negative	n/a	mixed	n/a
Fujii 2006	static	negative	n/a	n/a	n/a	n/a
Durell et al. 2006	static	negative	negative	n/a	negative	n/a
Stillman et al. 2005	dynamic	negative	mixed	n/a	mixed	n/a
Austin and Rehfisch 2003	dynamic	mixed	mixed	mixed	mixed	mixed

Table S4. Number of Western Hemisphere Shorebird Reserve Network (WHSRN) sites of hemispheric, international, and regional importance located within each country in the network (from WHSRN 2019).

Country	Number of WHSRN sites			
	Hemispheric	International	Regional	Total
North America				
Canada	3	1	3	7
United States	12	15	22	49
Mexico	3	6	11	20
Caribbean				
The Bahamas	-	-	1	1
Bonaire (the Netherlands)	-	-	1	1
Puerto Rico	-	-	1	1
Central America				
Costa Rica	-	-	1	1
Honduras	-	-	1	1
Nicaragua	-	1	-	1
Panama	1	1	-	2
South America				
Argentina	2	4	5	11
Bolivia	-	-	1	1
Brazil	1	1	2	4
Chile	2	1	7	10
Colombia	-	-	2	2
Ecuador	-	1	2	3
Paraguay	-	-	1	1
Peru	-	-	4	4
Suriname	3	-	-	3
Uruguay	-	-	1	1
Venezuela	-	1	-	1
